# Curing regime controlled performance of basalt-modified alkali activated slag: phase composition, porosity profile, microstructure and compressive strength

**DOI:** 10.1039/d6ra07623h

**Published:** 2026-10-14

**Authors:** Musa A. Said, M. Ramadan, Fatma. M. Helmy, Hany M. Abd El-Lateef, Mai M. Khalaf, Mohammed A. O. Basubaih, Saad Shaaban, Nagih M. Shaalan, Abdullah A. Saber, Alaa Mohsen

**Affiliations:** a Department of Chemistry, Faculty of Science, Islamic University of Madinah Madinah 42351 Saudi Arabia; b Department of Chemistry, Faculty of Science, Ain Shams University Cairo Egypt m.ramadan@sci.asu.edu.eg; c Department of Chemistry, College of Science, King Faisal University Al-Ahsa 31982 Saudi Arabia hmahmed@kfu.edu.sa; d Department of Physics, College of Science, King Faisal University Al-Ahsa 31982 Saudi Arabia; e Department of Biology, College of Science, Imam Mohammad Ibn Saud Islamic University (IMSIU) Riyadh 11623 Saudi Arabia; f Faculty of Engineering, Ain Shams University Cairo Egypt

## Abstract

The transition from traditional cement to low-carbon alkali-activated materials is no longer an environmental obligation but a materials-design challenge requiring precise control of reaction kinetics. In this study, basalt-modified alkali-activated slag was cured under various conditions to demonstrate how mix design and curing regimes affect gel formation and strength development. Ground-granulated blast-furnace slag (GGBFS) was replaced with 5–20 wt% basalt (BT) and then activated with 6 wt% NaOH. The developed materials were cured under normal conditions (1–28 days), hydrothermal conditions (135–170 °C/5 h) and microwave conditions (450 W/5–15 min). Advanced microstructural analyses (XRD, FTIR, SEM/EDX and BET/BJH) elucidated the phase evolution, morphological features and porosity profile within the matrices. The results reveal that BT has a slight effect on the fresh properties (workability and setting time) of the alkali-activated slag. Despite diluting reactive calcium phases in GGBFS, the AAS-20BT (80% GGBFS + 20% BT) composite delivered robust, structural-grade compressive strengths: 49.5 MPa under normal curing, 56 MPa after 170 °C/5 h hydrothermal treatment, and a rapid 37 MPa after just 10 min at 450 W of microwave exposure. From the compressive-strength results and advanced analysis techniques, it can be concluded that the curing regime strongly controls the composition of the load-bearing capacity phases, microstructural development and pore refinement. Overall, hydrothermal curing provides the most favorable balance between reaction kinetics, zeolitic phase formation, hydrate morphology and microstructural development. Under normal conditions, BT particles act as fillers and nucleation seeds, while hydrothermal curing increases their reactivity, facilitating Si and Al dissolution and participation in geopolymeric products. Accordingly, the AAS-20BT can be considered a promising low-carbon binder that offers a highly scalable, eco-efficient pathway for next-generation construction materials.

## Abstract 2

Basalt–slag alkali-activated binders were developed and subjected to ambient, autoclave, and microwave curing to achieve rapid hardening for precast applications.

## Graphical abstract

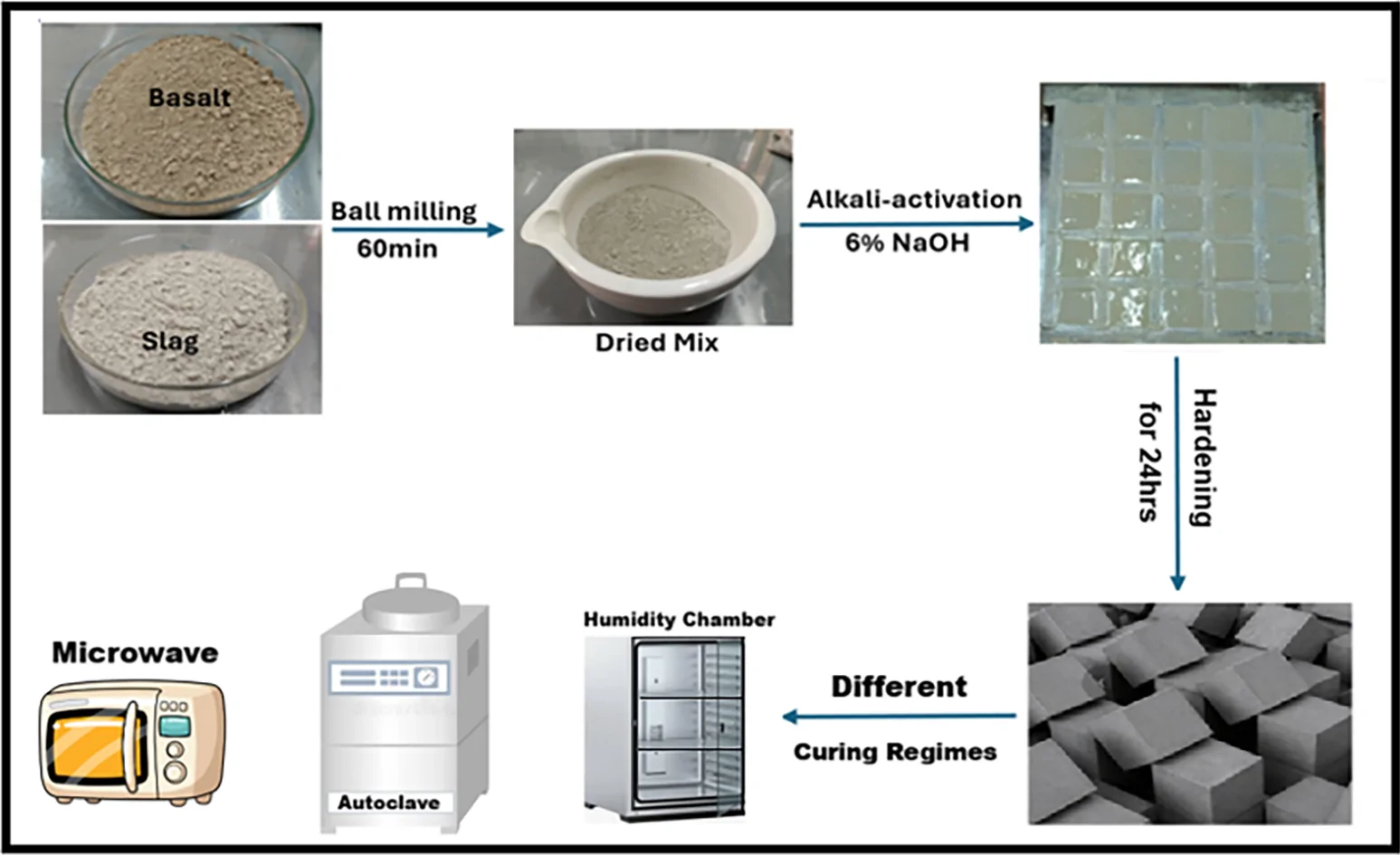

## 1. Introduction

The environmental burden associated with ordinary Portland cement (OPC) has driven extensive research toward the development of alternative binders, among which alkali-activated materials and geopolymers have gained significant attention. These materials are synthesized through the activation of aluminosilicate precursors, offering reduced carbon emissions and enhanced sustainability compared to conventional cementitious systems.^1,2^ Ground granulated blast furnace slag (GGBFS) has been widely utilized in geopolymer formulations due to its high calcium content and latent hydraulic properties. Numerous studies have demonstrated that slag-based geopolymers exhibit rapid strength development and dense microstructures. However, the performance of slag-rich systems is highly dependent on curing conditions and activator composition, which influence the reaction kinetics and phase assemblage.^3,4^ In contrast, basalt, as a naturally abundant aluminosilicate material, has recently emerged as a potential precursor or supplementary material in geopolymer systems. Basalt is characterized by its chemical stability, availability, and relatively high silica and alumina content. Previous studies have indicated that basalt incorporation can enhance durability, thermal resistance, and long-term performance, although its relatively low reactivity compared to slag remains a challenge.^5,6^ The combined use of slag and basalt has been explored to leverage the advantages of both materials. Slag contributes to early strength and reactivity, while basalt enhances structural integrity and durability. Some researchers have reported that hybrid systems based on slag and basalt exhibit improved mechanical properties and resistance to aggressive environments, suggesting a synergistic interaction between the two materials.^7–11^ Recent advances in alkali-activated slag/basalt systems have demonstrated the potential of waste basalt powder as a sustainable aluminosilicate precursor for the development of high-performance composites. Shen *et al.* (2026) developed a high-performance alkali-activated basalt/slag composite exhibiting exceptional compressive strength and ductility. Their study showed that the simultaneous activation of slag and basalt powder led to the formation of a complex binder system consisting of both C-(N)-A-S-H and N-A-S-H gels. Moreover, significant interfacial ion interactions promoted the precipitation of Fe–Al and Mg–Al layered double hydroxide (LDH) phases, which enhanced the matrix microstructure and micromechanical performance.^12^

Curing conditions play a critical role in the geopolymerization process and significantly affect the final properties of the material. Conventional (ambient) curing is the most practical method for large-scale applications; however, it often results in slower reaction rates and lower early strength, particularly for low-calcium systems.^13,14^ Hydrothermal curing has been investigated as an effective technique to enhance geopolymerization by providing elevated temperature and pressure conditions. This method promotes the dissolution of aluminosilicate species and accelerates the formation of binding phases, leading to improved mechanical strength and microstructural refinement. Several studies have reported that hydrothermal treatment enhances the crystallinity and stability of geopolymer matrices.^15,16^

Microwave curing has also attracted attention as an advanced and energy-efficient curing technique. Unlike conventional heating, microwave curing provides rapid and volumetric heating, which significantly accelerates reaction kinetics. Research findings indicate that microwave-assisted geopolymerization can lead to shorter curing times, enhanced early strength, and more homogeneous microstructures. However, issues related to thermal gradients and potential microcracking require careful optimization.^10,17,18^

Despite the growing body of literature on slag-based geopolymers and alternative curing techniques, limited studies have systematically investigated the combined use of slag and basalt under different curing regimes, particularly when comparing conventional, hydrothermal, and microwave curing methods within a unified experimental framework. Moreover, the interaction between material composition and curing technique remains insufficiently understood, highlighting the need for further comprehensive investigations. Recent studies have investigated the partial replacement of slag with basalt powder or fibers in cementitious and geopolymer composites under varied curing regimes, yielding significant enhancements in mechanical properties and microstructural densification. Rashad *et al.* (2025) found that replacing 10–20% slag with Egyptian basalt powder under normal curing (28 days at 20–25 °C) achieved compressive strengths of 41.2–43.9 MPa, attributed to refined pore structures and increased SiO_2_ reactivity, with optimal performance below 20% to avoid excess unreacted particles.^19^ Similarly, Liu *et al.* (2025) reported that incorporating ground granulated blast furnace slag (GGBS) into recycled aggregate concrete, together with other supplementary materials such as copper slag and basalt fiber, improved the mechanical performance of the mixes. The enhancement was attributed to the densifying effect of GGBS, which reduced internal voids and contributed to a stronger microstructure.^20^ Sadrmomtazi *et al.* (2018) examined basalt fiber reinforced cementitious composites under hydrothermal treatment and reported that this condition strengthened fiber–matrix bonding, leading to improved flexural performance and better resistance to microcrack propagation,^21^ while Jing *et al.* (2008) demonstrated tobermorite (Ca_5_Si_6_O_16_(OH)_2_·4H_2_O) formation in slag systems with mineral replacements after 48 h at 200 °C, enhancing early hydration.^22^ Sünbül and Tortum (2025) reported that the incorporation of 25% basalt nano-powder under ultrasonic treatment enhanced the compressive strength of hybrid cementitious systems, indicating the potential of basalt-derived nanomaterials in improving mechanical performance and sustainability.^23^ In a related study, Rashad *et al.* (2025) found that using 20% Egyptian basalt powder in alkali-activated slag cement significantly improved durability under wetting–drying cycles, reducing strength loss and promoting a denser pore structure through filler effects and secondary N-A-S-H gel formation.^19^ In addition, Aschoff *et al.* (2024) demonstrated that microwave curing can accelerate the hardening of geopolymer systems by promoting internal heating, moisture removal, and rapid polycondensation, which leads to faster strength development and early-age performance.^24^

The motivation of the present study stems from the need to develop sustainable geopolymer systems with enhanced performance by utilizing both industrial by-products and natural resources. While previous studies have provided valuable insights into slag-based geopolymers and advanced curing techniques, there remains a lack of integrated studies addressing the combined effect of slag and basalt under multiple curing conditions. This research distinguishes itself by systematically evaluating the synergistic interaction between slag and basalt as replacement materials within geopolymer matrices, while simultaneously comparing the influence of normal, hydrothermal, and microwave curing techniques on the compressive strength, phase composition and microstructural properties. Such a comprehensive approach enables a deeper understanding of the governing mechanisms and provides a basis for optimizing sustainable construction materials suitable for practical applications. Therefore, this study not only contributes to reducing the environmental impact of cement production but also advances the current state of knowledge by bridging the gap between material composition and curing methodology in geopolymer technology.

## 2. Materials and methodology

### 2.1. Materials

This research focuses on the application of two kinds of raw materials; these materials include highly amorphous waste slag (GGBFS) delivered from Lafarge Company in Ain El-Sokhna. Egyptian basalt (BT) was obtained from Sinai Cement Company in El-Arish. It is important to note that GGBFS is considered a primary by-product of the steel industry while BT is a naturally occurring aluminosilicate rock that can be used as a potential precursor for geopolymer synthesis. Commercial-grade sodium hydroxide (NaOH), which served as an alkaline activator, was supplied by α-Aromatic Company in Cairo, Egypt. Table 1 shows the chemical-oxide composition of GGBFS, and BT. XRF analysis of GGBFS indicates it is a calcium-rich aluminosilicate precursor, with CaO, SiO_2_, and Al_2_O_3_ at 24.50, 50.01, and 14.11 wt%, respectively, showing its hydraulic reactivity. BT matches GGBFS in SiO_2_ and Al_2_O_3_ content, at 48.83 and 14.30 wt%. Additionally, BT contains significant amounts of Fe_2_O_3_ (16.40 wt%). The low loss on ignition (LOI) confirms low moisture content and minimal carbonate and organic material presence. Ultimately, XRF analysis demonstrates that BT is aluminosilicate precursors suitable for use in blended cement and geopolymeric composites.

**Table 1:** XRF analysis to identify oxide compositions of utilized raw materials (mass%)

Materials	SiO_2_	Al_2_O_3_	Fe_2_O_3_	CaO	MgO	TiO_2_	SO_3_	Na_2_O	K_2_O	Cl	LOI
GGBFS	50.01	14.11	0.78	24.50	6.32	—	1.78	0.52	0.86	0.61	0.51
BT	48.38	14.30	16.40	11.80	2.85	2.03	0.23	1.58	1.18	0.11	0.31

The XRD-patterns of raw materials are shown in Fig. 1. It was conducted with Cu-target at 30 mA and 40 kV (*λ* = 0.15418 nm); the scanning range was 2*θ* = 5–60° with a scanning speed of 0.02° s^−1^. GGBFS displays a distinctive broadband, indicating its semi-crystalline nature (44.32%). It contains some small reflections associated with gehlenite, akermanite, and calcite. BT exhibits a high degree of crystallinity (82.8%), primarily composed of anorthite, labradorite, augite, and magnetite. The particle size distribution (PSD, Fig. 2) shows a significant difference in sizes between the utilized materials. The results demonstrate that GGBFS has (D50, Dmax and dominant size of 1.26, 11.67 and 0.85 µm), reflecting its high reactivity. The PSD of BT clarifies that it has the finest particle size (D50, Dmax and dominant size of 0.32, 2.96 and 0.20 µm). The morphology of the raw precursors used in this study was examined using SEM, as shown in Fig. 3. GGBFS (Fig. 3a1 and a2) reveal that it consists of irregular and angular particles. While BT (Fig. 3b1 and b2) display densely packed, blocky-crystal, and smooth-cleavage particles.

**Fig. 1:**
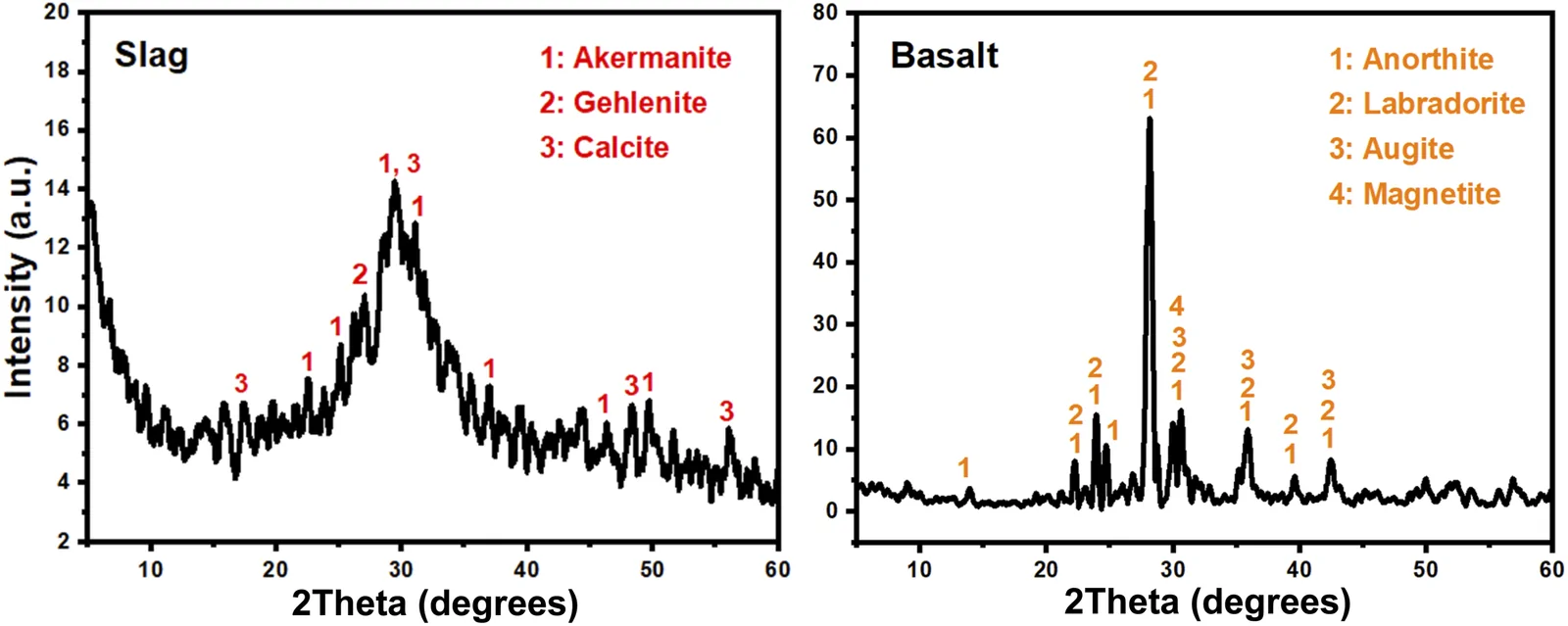
XRD-patterns of raw materials.

**Fig. 2:**
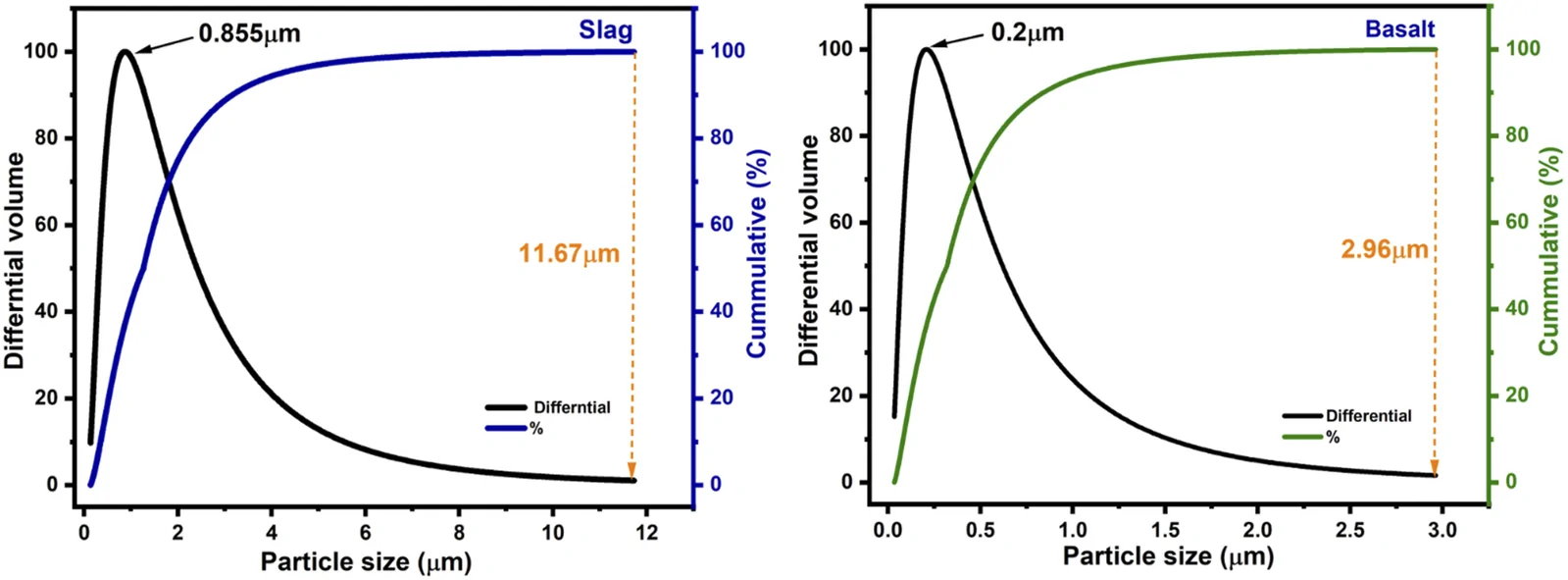
Particle size distribution curves of raw materials.

**Fig. 3:**
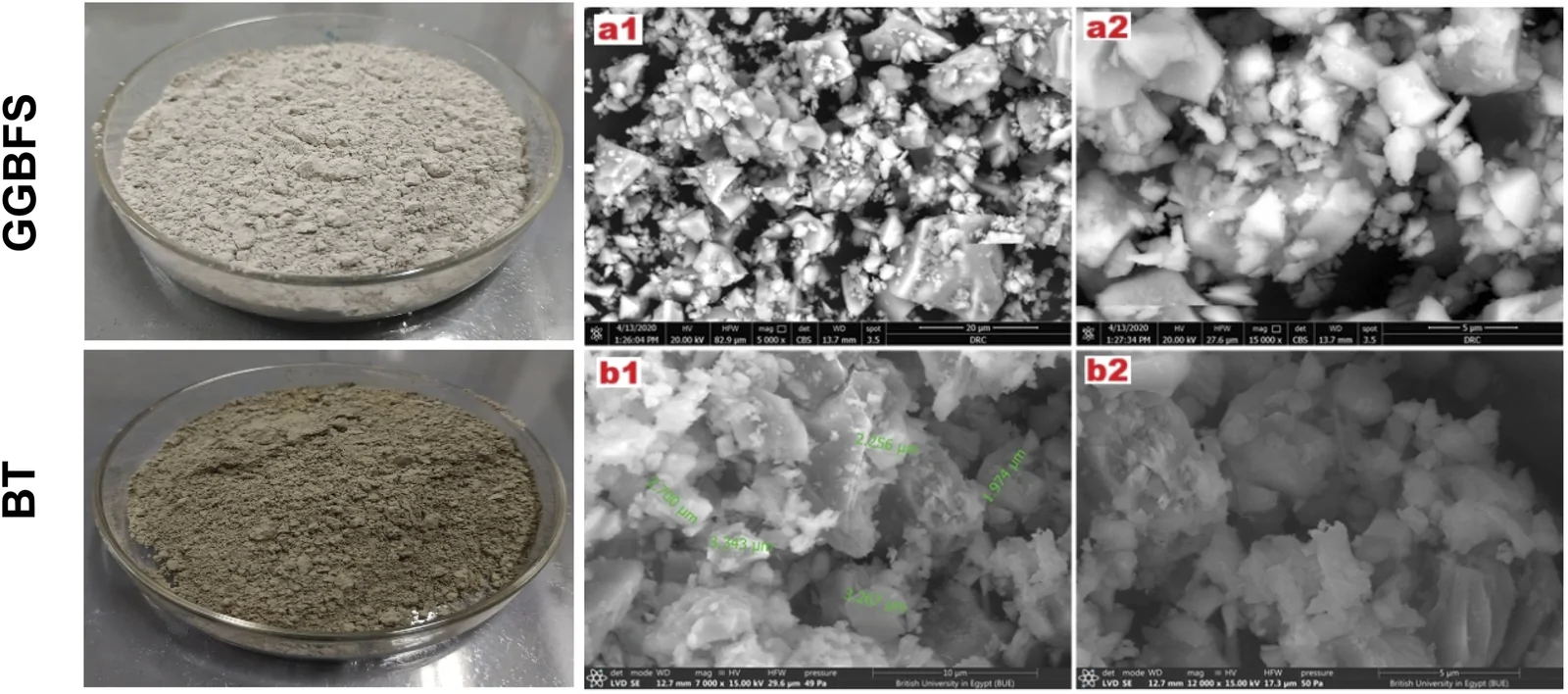
Real and SEM images for: (a1 and a2) GGBFS and (b1 and b2) BT.

### 2.2. Methodology

#### 2.2.1. Casting of fresh pastes

Four alkali-activated composite fresh pastes were made *via* NaOH activation of slag and basalt, as shown in Table 2. The concentration of NaOH was 6% by mass of total binders; this concentration was chosen based on a preliminary study of mechanical efficiency in addition to economic and environmental considerations, as reported in previous publications.^25–28^ The first mixture (AAS), considered the reference, was made of 100% slag, equivalent to 800 g. The second dry mixture (AAS-20BT) contained 80% slag (640 g) + 20% basalt (160 g). The third dry mixture (AAS-10BT) contained 90% slag (720 g) + 10% basalt (80 g). The fourth dry mixture (AAS-5BT) contained 95% slag (760 g) + 5% basalt (40 g). Each dry mixture was well mixed using a porcelain ball mill for 60 min. Next, every dry mixture was activated using an alkaline aqueous solution containing 48 g of sodium hydroxide dissolved in 240 mL of pouring water, as water/binder (W/B = 0.3). This ratio was detected based on the Vicat apparatus (ASTM C1161-18),^29^ indicating the appropriate flowability. An ELE-automatic mixer was used to obtain a workable paste *via* mixing the dry samples with the aqueous alkaline solution for 3 min. Four alkali-activated composite fresh pastes (AAS, AAS-20BT, AAS-10BT, AAS-5BT) were individually prepared then cast in cubic stainless-steel mold (25 × 25 × 25 mm).^30^ A vibrating table was used to remove bubbles and to obtain tight pastes after the hardening process. The cast pastes were initially cured for 24 h under ambient conditions at high relative humidity to ensure adequate setting and early hardening. Following this initial curing period, the alkali-activated specimens were separated into three curing groups. The first group was maintained under conventional high-humidity conditions for 1, 3, 7, and 28 days. The second group was subjected to hydrothermal curing in an autoclave for 5 h at temperatures of 135, 155, and 170 °C. The third group underwent microwave curing for 10 min at a power level of 450 W. These curing parameters were selected based on recommendations and findings reported in previous studies.^27,28,31–33^

**Table 2:** Mix design and codes of geopolymeric pastes

Mix code	GGBFS%	BT%	W/S ratio%	NaOH%
AAS	100	—	0.3	6
AAS-5BT	95	5	0.3	6
AAS-10BT	90	10	0.3	6
AAS-20BT	80	20	0.3	6

#### 2.2.2. Physico-mechanical testing and characterization

The workability of the freshly prepared geopolymeric pastes (AAS, AAS-5BT, AAS-10BT, and AAS-20BT) was evaluated using a modified mini-slump test. The flow behavior was quantified by measuring the spread diameter, which was subsequently used to calculate the corresponding spread area. A larger spread area was considered indicative of enhanced flowability and workability of the fresh paste systems.^34^ The setting characteristics, including both initial and final setting times, were determined using a Vicat apparatus in accordance with the procedures specified in ASTM C191.^35^ The workability and setting time tests were conducted on three independent specimens for each mixture, and the reported values correspond to the arithmetic mean of the obtained results. The variability of the measurements was evaluated through the standard deviation, which ranged from 2.0 to 2.7 cm^2^ for workability and from 2.0 to 4.0 min for setting time. Error bars representing ± one standard deviation were included in the corresponding figures to illustrate the repeatability and experimental variability of the measurements.

The mechanical performance of the hardened composites was assessed through compressive strength testing on four cubic specimens per mix under the various curing conditions, following ASTM C109M (2013).^36^ The tests were conducted using a Ton-Industrie compression testing machine with a maximum loading capacity of 60 tons. For the compressive strength evaluation, four cube specimens were tested for each mixture and curing condition, and the reported values represent the average compressive strength of the four specimens. The corresponding standard deviations ranged from 2.0 to 2.5 MPa for ambient curing, 1.8 to 3.0 MPa for hydrothermal curing, and 1.8 to 2.6 MPa for microwave curing. Error bars corresponding to ±one standard deviation were incorporated into the strength figures to provide a quantitative indication of the experimental scatter and reproducibility of the test results under the different curing regimes.

To gain insight into the physicochemical properties and microstructural evolution of the synthesized alkali-activated pastes, selected specimens were subjected to comprehensive characterization techniques. X-ray diffraction (XRD) was employed to identify the crystalline and amorphous phase assemblages, while Fourier-transform infrared spectroscopy (FTIR) was utilized to investigate the structural evolution and functional groups associated with the reaction products. The pore structure and specific surface characteristics were analyzed using Brunauer–Emmett–Teller/Barrett–Joyner–Halenda (BET/BJH) methods, whereas scanning electron microscopy coupled with energy-dispersive X-ray spectroscopy (SEM/EDX) was performed to examine the morphology, elemental composition, and distribution of the formed binding hydrates. A schematic diagram of the experimental work is represented in Fig. 4.

**Fig. 4:**
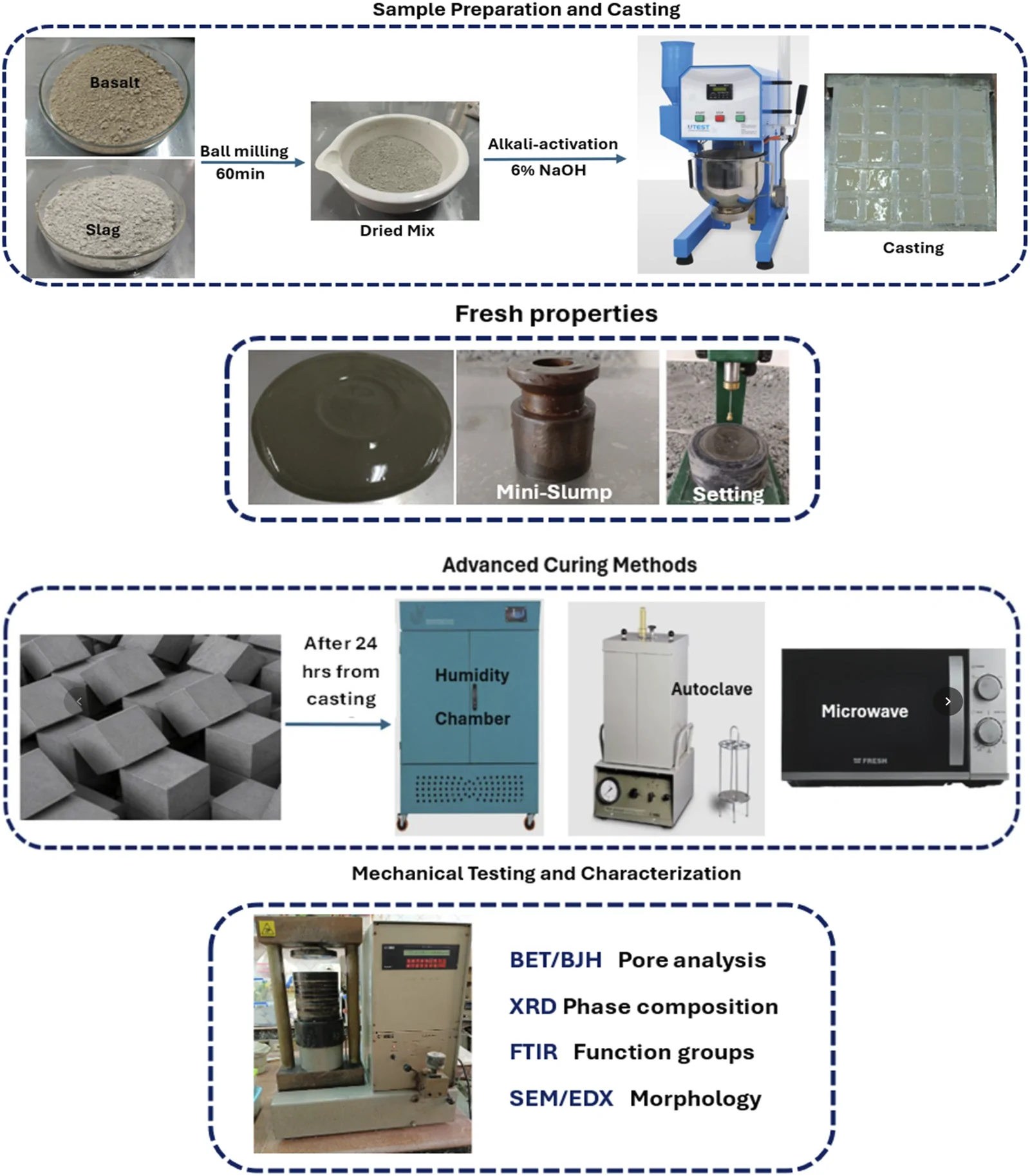
A schematic diagram shows the whole experimental program.

## 3. Results and discussion

### 3.1. Fresh properties

In cementitious materials and geopolymeric binders, workability and setting time are the two main fresh state indicators that govern their applicability to be used in the construction sector.^37^ They determine the ease of mixing, transporting, placing, and compacting before a rigid load-bearing skeleton develops.^38,39^ Accordingly, the workability and initial/final-setting time (I/F-ST) for the developed geopolymeric composites (AAS, AAS-5BT, AAS-10BT, and AAS-20BT) were recoded. Fig. 5 shows a clear sequence in workability; the largest spreading area corresponds to the highest workability. The spreading area values of AAS, AAS-5BT, AAS-10BT, and AAS-20BT were 83.3, 82.6, 78.5, and 73.9 cm^2^, respectively. Compared with the control mix (AAS), the spreading area decreased by only 0.84%, 5.76%, and 11.28% for AAS-5BT, AAS-10BT, and AAS-20BT, respectively, indicating a slight but progressive reduction in workability with increasing BT content. This behavior can be attributed to the markedly finer particle size distribution of BT (D50 = 0.32 µm) compared with GGBFS (D50 = 1.26 µm).^40,41^ The ultrafine BT particles provide a higher specific surface area, resulting in increased adsorption of the alkaline activator solution and a corresponding reduction in the amount of free liquid available for flow. Consequently, the fresh mixtures exhibited a gradual decrease in spreading area as the BT replacement level increased. Nevertheless, the observed reduction remained relatively limited, indicating that the incorporation of BT exerted only a minor influence on the workability of the alkali-activated mixtures.^37^

**Fig. 5:**
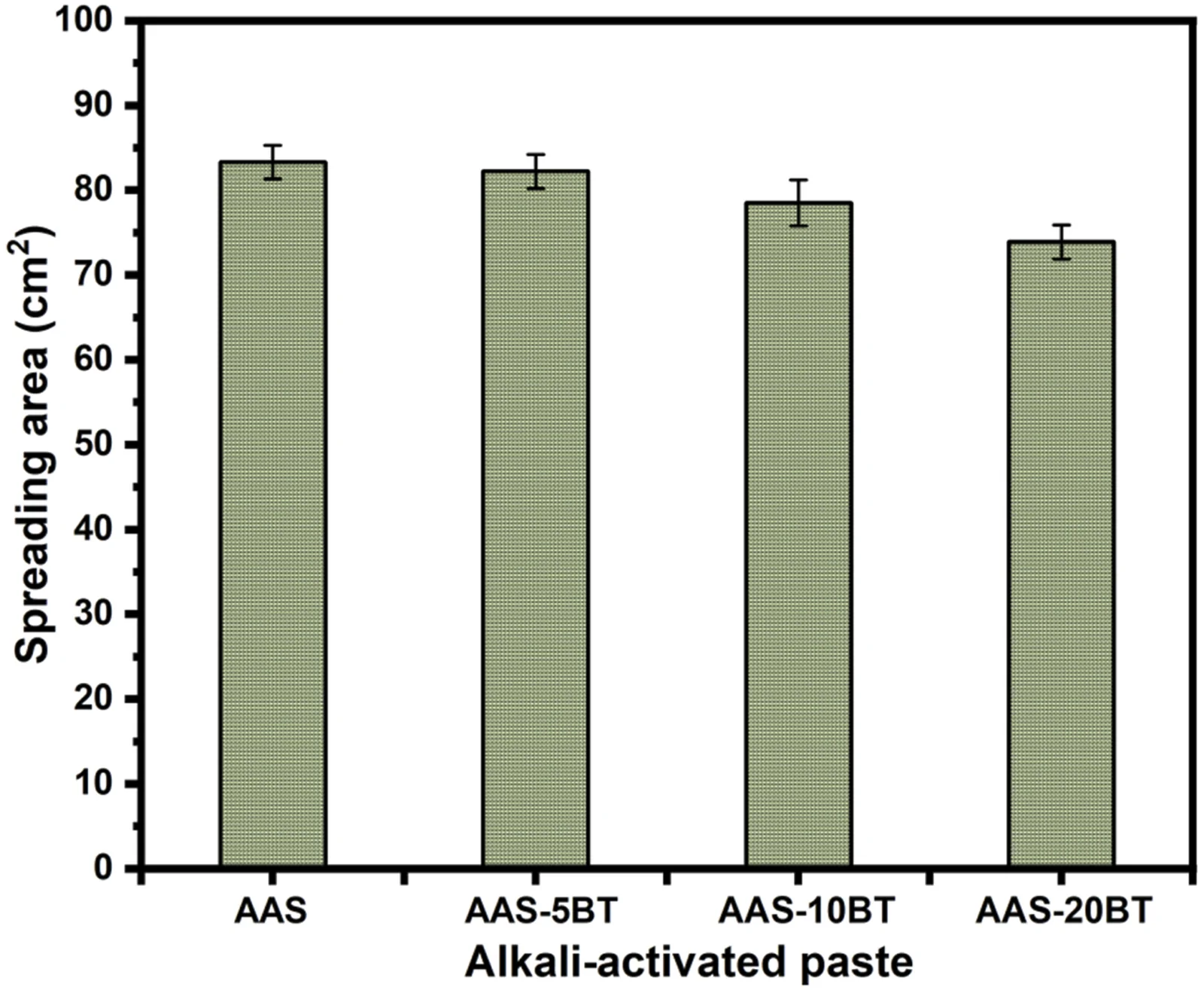
Workability of fresh alkali-activated slag pastes containing different ratios of basalt.

For the setting time (Fig. 6), the AAS, AAS-5BT, AAS-10BT, and AAS-20BT pastes exhibited initial/final setting times (I/F-ST) of 82/104, 78/100, 80/98, and 90/108 min, respectively. Notably, the I/F-ST values of all AAS-BT blends fall within the acceptable range for binding materials, confirming their suitability for construction applications.^42^ Furthermore, the setting times of AAS-5BT and AAS-10BT are comparable to those of the reference AAS paste, indicating that the incorporation of basalt tailings up to 10% does not significantly affect the setting behavior of the alkali-activated slag system. However, a slight increase in both the initial and final setting times was observed for AAS-20BT. Increasing the BT content up to 20% alters the chemical composition of the precursor system, resulting in lower overall CaO content and consequently reducing the availability of calcium required for the formation of early strength-developing reaction products. This reduction in calcium availability can hinder the precipitation of calcium silicate hydrates (CSH), calcium aluminosilicate hydrates (CASH), and calcium aluminate hydrates (CAHs) gels, thereby slowing the hardening process and extending the setting time. Accordingly, the prolonged setting time observed for AAS-20BT is primarily attributed to the dilution of the calcium-rich GGBFS phase rather than to any significant change in the SiO_2_/Al_2_O_3_ ratio, which remains nearly unchanged at the applied basalt replacement level.^43,44^

**Fig. 6:**
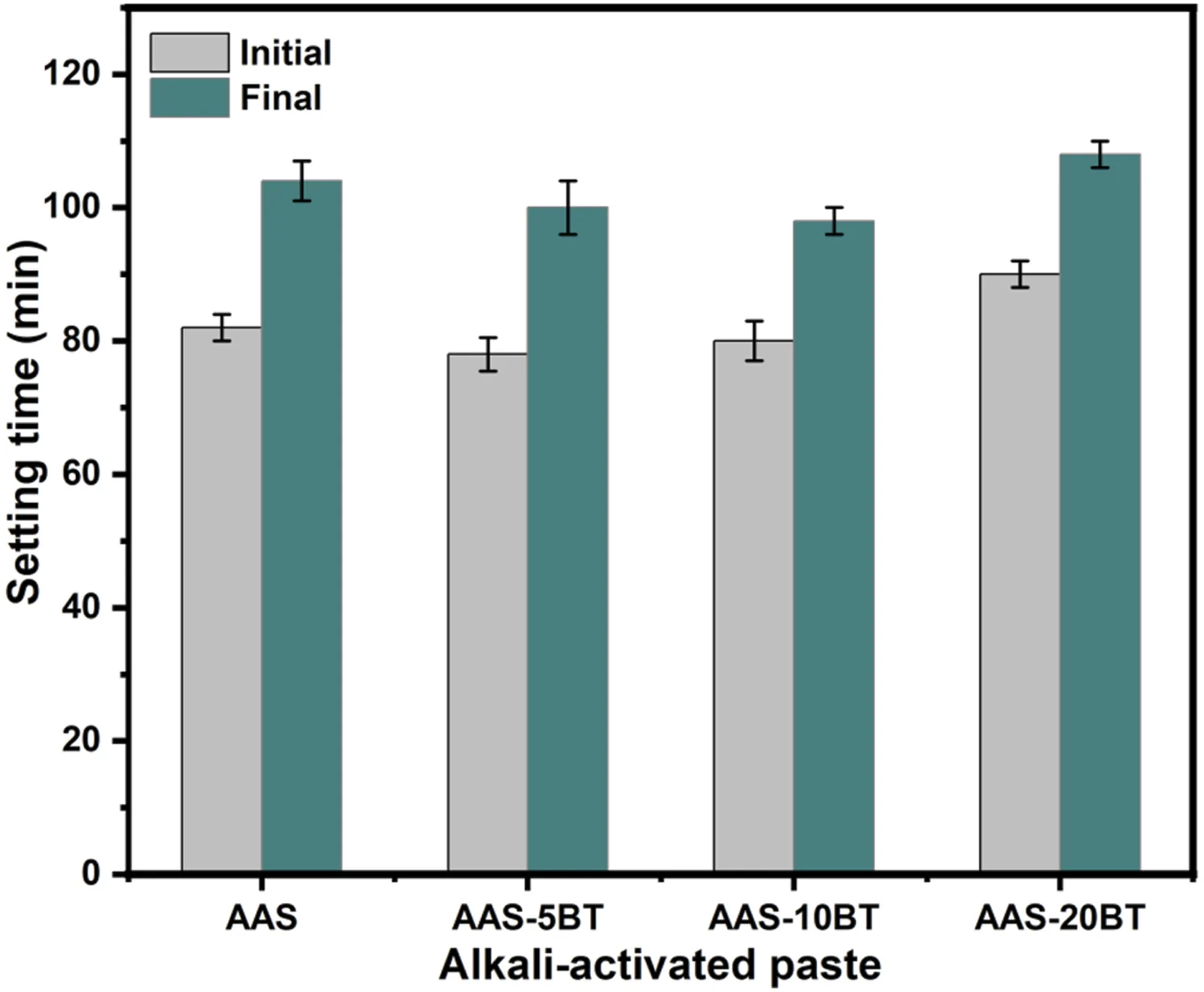
Setting times of fresh alkali-activated slag pastes containing different ratios of basalt.

### 3.2. Compressive strength

The impact of basalt content (0, 5, 10 and 20 wt% concerning AAS) on the compressive-strength of the normally cured AAS–basalt composites at 1, 3, 7 and 28 days is represented in Fig. 7. Generally, it is detected that all fabricated composites exhibit significant improvement in the compressive-strength as the curing process continued.^45^ This enhancement can be associated with the ongoing hydration and geopolymerization of the slag-based system, which leads to the formation of binding phases such as CSH, C(A)SH, CAH, and hydrotalcite-like phases. The progressive precipitation of these products around slag and basalt particles and within the pore network refines the microstructure, decreases pore connectivity, and improves the mechanical properties.^46–49^Fig. 7 demonstrates that increasing the basalt (BT) content leads to a reduction in compressive strength, particularly at early curing ages. At 1 day, the compressive strengths of AAS, AAS-5BT, AAS-10BT, and AAS-20BT were 41.6, 27.0, 20.2, and 17.9 MPa, respectively. The lower early-age strength of the BT-containing mixtures can be attributed to the partial replacement of the highly reactive slag by the less reactive basalt tailings, which reduces the availability of reaction products responsible for rapid matrix development during the initial stages of alkali activation. Consequently, the formation of the principal binding phases identified by XRD (see Fig. 12), including C-(A)-S-H, C-A-H, and hydrotalcite-like phases, is delayed, resulting in slower early strength development. Nevertheless, continuous curing significantly enhanced the compressive strength of all mixtures. After 28 days, the compressive strengths reached 73.1, 63.0, 54.4, and 49.4 MPa for AAS, AAS-5BT, AAS-10BT, and AAS-20BT, respectively. Although the AAS-20BT mixture exhibited a strength reduction of 56.97% relative to the reference mix at 1 day, this reduction decreased markedly to 32.42% after 28 days, highlighting the continued contribution of BT-containing systems to strength development at later ages and densification of the reaction products, as well as the filler and nucleation effects of basalt particles, which contribute to a more compact microstructure and partial compensation for the initial dilution effect.^46–48^

**Fig. 7:**
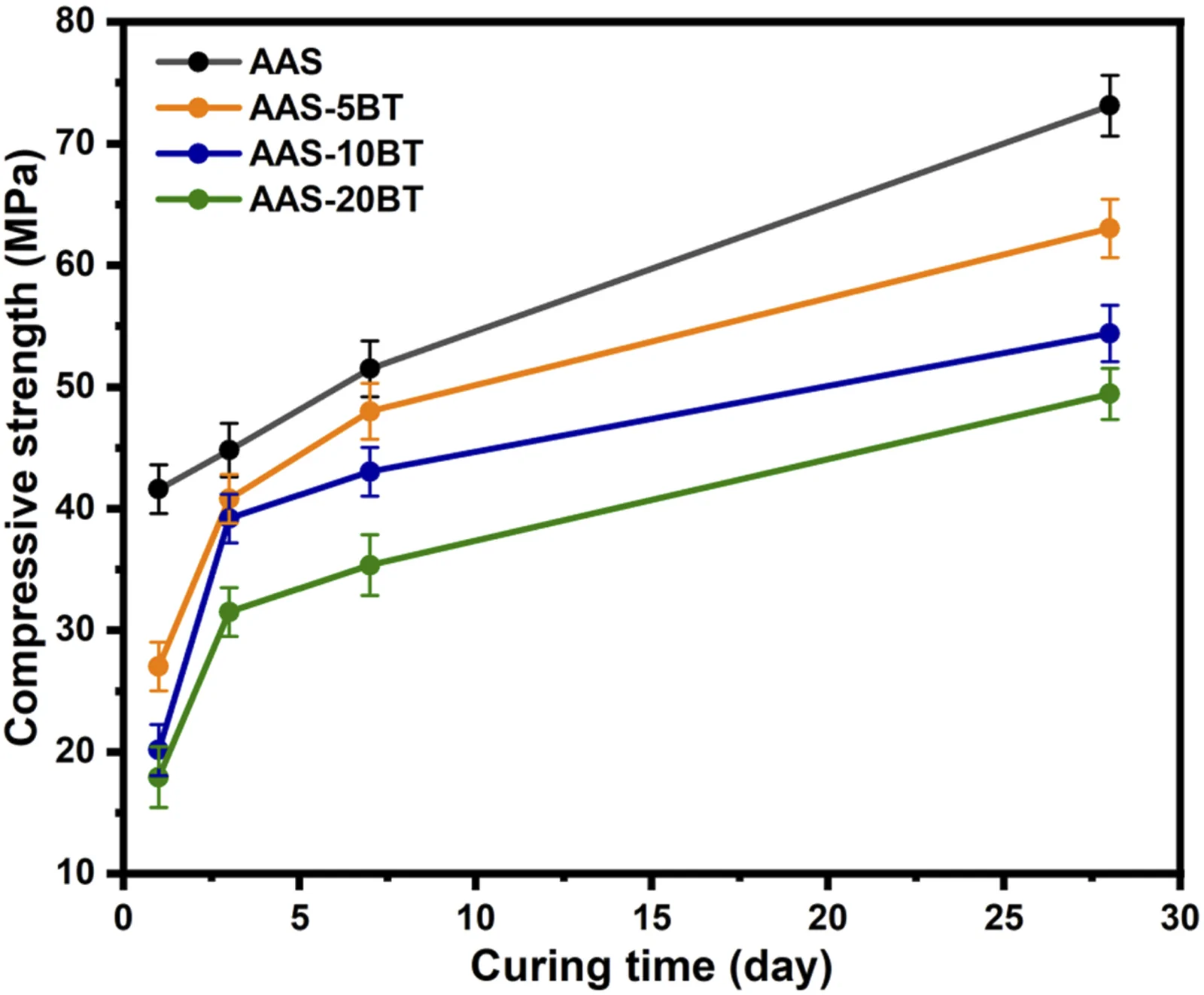
Compressive strength values of AAS and AAS-BT composite pastes up to 28-days of normal curing.

To investigate the feasibility of producing precast building materials from the developed composites, Fig. 8 illustrates the influence of hydrothermal treatment (autoclave curing) at 135, 155, and 170 °C for 5 h on the compressive strength of alkali-activated slag–basalt pastes. In general, increasing the hydrothermal curing temperature enhanced the mechanical performance of all mixtures, although the magnitude of improvement varied with basalt content.

**Fig. 8:**
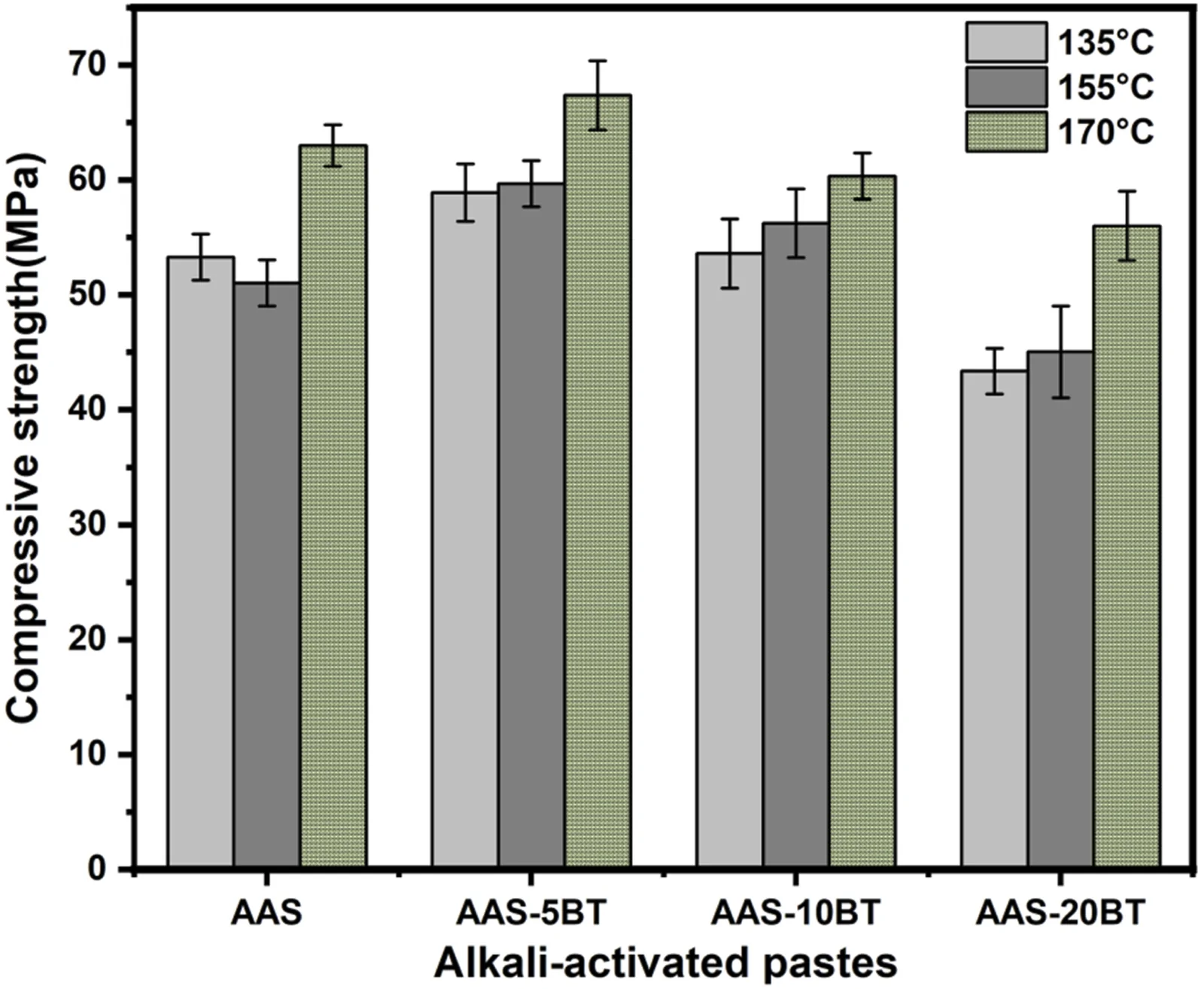
Compressive strength values of AAS and AAS-BT composite pastes under autoclave curing at different temperatures for 5 h.

The highest compressive strengths were consistently achieved at 170 °C, reaching 63, 67.4, 60.3, and 56 MPa for AAS, AAS-5BT, AAS-10BT, and AAS-20BT, respectively. The superior performance at 170 °C can be attributed to the acceleration of precursors (slag/basalt) dissolution and reaction kinetics under more severe hydrothermal conditions, leading to enhanced formation and crystallization of strength-contributing reaction products.^50^ Elevated temperature promotes the transformation of the initially formed amorphous hydrates gel into more compact and crystalline calcium aluminosilicate hydrate, hydrogarnet, and zeolitic phases (as detected *via* XRD), resulting in improved matrix densification and reduced porosity.^27,34,45,51^ Furthermore, hydrothermal treatment facilitates particle rearrangement and pore refinement (affirmed *via* BET/BJH model (Fig. 10)), producing a denser microstructure and stronger interparticle bonding. Consequently, all mixtures exhibited their optimum mechanical performance after autoclave curing at 170 °C. Among the investigated mixtures, AAS-5BT exhibited the highest compressive strength under all hydrothermal curing conditions. Increasing the curing temperature from 135 to 170 °C enhanced the compressive strength of all mixtures by approximately 18.25%, 14.41%, 12.54%, and 29.15% for AAS, AAS-5BT, AAS-10BT, and AAS-20BT, respectively. These results suggest that elevated hydrothermal temperatures are beneficial for strength development, with the most pronounced enhancement observed for AAS-20BT.

To further explore rapid curing alternatives, the influence of microwave irradiation at 450 W for 5, 10, and 15 min on the compressive strength of alkali-activated slag–basalt pastes was investigated, as shown in Fig. 9. In general, extending the microwave treatment time from 5 to 10 min improved the compressive strength of all mixtures, indicating that moderate microwave exposure accelerated alkali-activation reactions and promoted the formation of binding phases.^52–54^ The highest compressive strengths were therefore achieved after 10 min of microwave curing, reaching 39.2, 42.9, 40, and 37 MPa for AAS, AAS-5BT, AAS-10BT, and AAS-20BT, respectively. Further increasing the irradiation time to 15 min resulted in a reduction in compressive strength for all mixtures. This behavior may be attributed to excessive moisture loss and the development of thermal stresses associated with prolonged microwave exposure, which can induce microcracking and adversely affect matrix densification.^55^

**Fig. 9:**
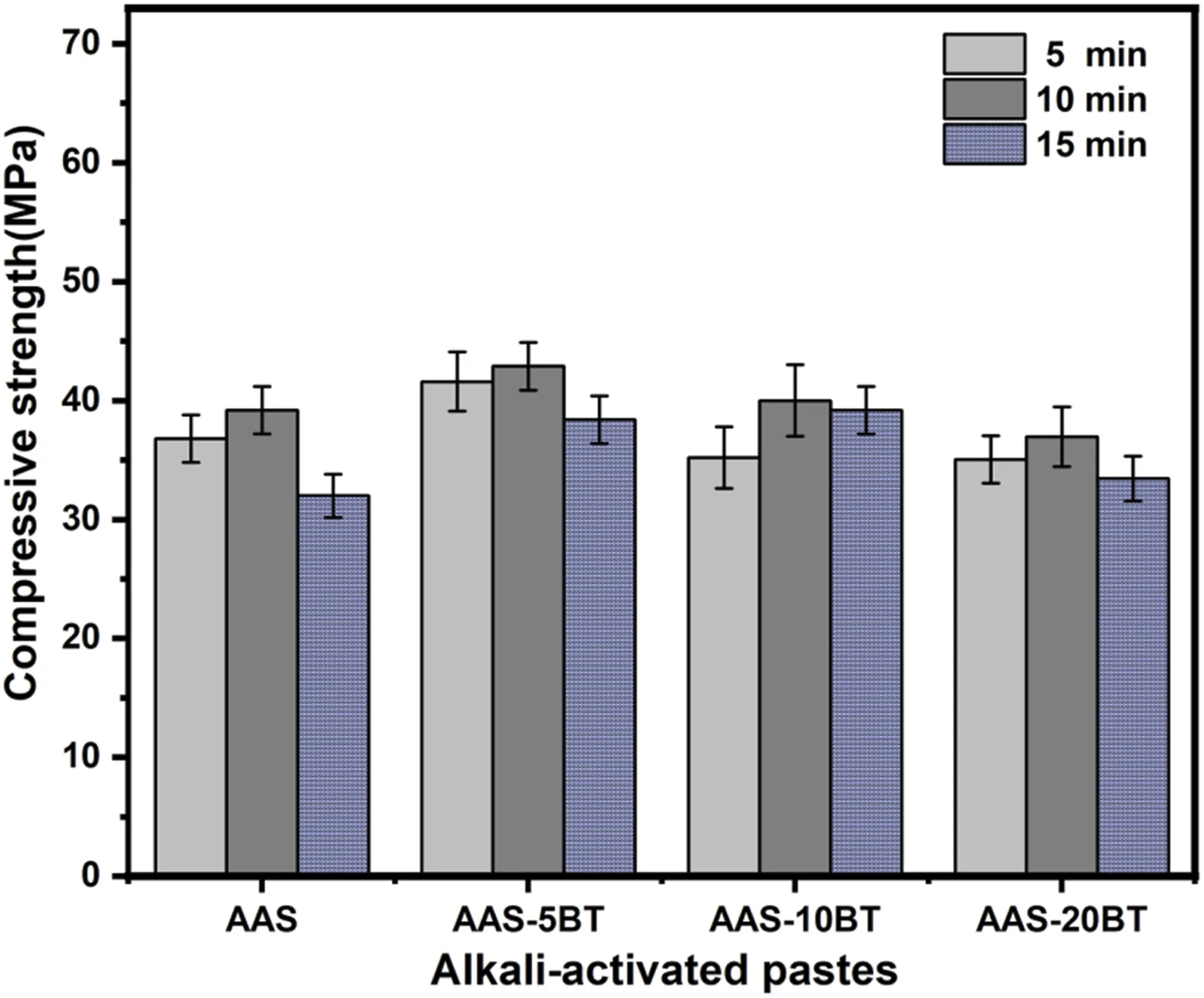
Compressive strength values of AAS and AAS-BT composite pastes under microwave treatment for 15 min at 450 W.

For the AAS-20BT mixture, the compressive strength under normal curing reached 49.4 MPa after 28 days, whereas microwave curing at 450 W for 10 min produced a lower strength of 37 MPa, corresponding to a reduction of approximately 25.2%. In contrast, hydrothermal curing at 170 °C for 5 h increased the compressive strength to 56 MPa, representing an improvement of about 13.4% relative to normal curing. These results suggest that hydrothermal treatment is more effective than microwave curing in enhancing the mechanical performance of AAS-20BT, likely because the elevated temperature and pressure promote more extensive reaction of the aluminosilicate precursors and the development of a denser binding matrix. Meanwhile, the rapid heating associated with microwave curing may not provide sufficient time for reaction-product growth and could contribute to moisture loss and microstructural defects, leading to lower strength values. To provide insight into the strength development mechanisms, the AAS and AAS-20BT pastes cured under different regimes were comprehensively characterized using XRD, FTIR, BET/BJH, and SEM/EDX techniques.

### 3.3. Pore structure analysis

The N_2_ adsorption/desorption isotherms and BJH pore size distribution curves of AAS and AAS-20BT pastes subjected to different curing regimes are presented in Fig. 10, while the corresponding textural parameters are summarized in Table 3. The obtained results reveal a strong dependence of pore structure development on the curing method and provide valuable insight into the mechanical performance of the studied systems. For the normally cured AAS paste (28 days), the adsorption isotherm exhibits a Type III profile, characterized by relatively weak adsorbate–adsorbent interactions and a gradual increase in nitrogen uptake at high relative pressures. The corresponding pore size distribution indicates the predominance of wide mesopores with an average pore diameter of 95.5 nm, accompanied by a relatively low pore volume (0.0397 cm^3^ g^−1^) and specific surface area (10 755 cm^2^ g^−1^). After 28 days of curing, the AAS paste attained the highest compressive strength among all ambient-cured mixtures (73.1 MPa). This high strength suggests that the continuous generation of CSHs, CASHs, CAHs, hydrotalcite-like phases during prolonged curing produced a dense and interconnected aluminosilicate matrix, despite the presence of relatively large mesopores detected by nitrogen adsorption.^7^

**Fig. 10:**
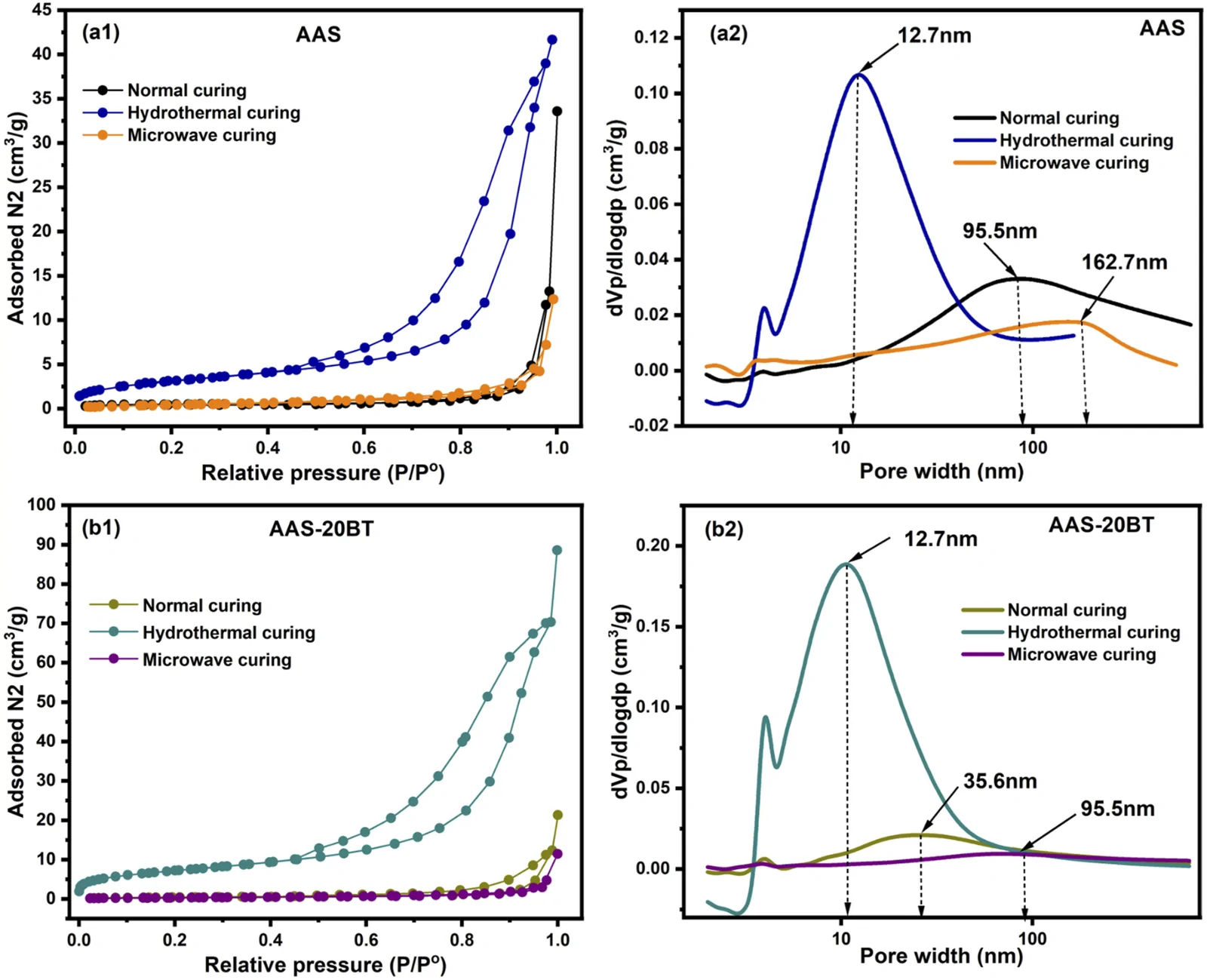
N_2_-adsorption/desorption isotherms and BJH-pore size distributions for: (a1 and a2) AAS and (b1 and b2) AAS-20BT.

**Table 3:** Textural parameters of AAS and AAS-20BT pastes at different curing regimes

Composite	Curing condition	SSA (cm^2^ g^−1^)	*V*_p_ (cm^3^ g^−1^)	dp_max_ (nm)	Isotherm type
AAS	Normal up to 28 days	10 755	0.0397	95.5	III
	Hydrothermal (170 °C/5 h)	170 260	0.0654	12.7	IV/H3
	Microwave (450 W/10 min)	28 432	0.0186	162.7	III
AAS-20BT	Normal up to 28 days	29 767	0.0241	35.6	III
	Hydrothermal (170 °C/5 h)	397 290	0.1168	12.7	IV/H3
	Microwave (450 W/10 min)	19 984	0.0136	95.5	III

When AAS paste was exposed to hydrothermal curing at 170 °C for 5 h, the adsorption behavior changed markedly, displaying a Type IV isotherm with an H3 hysteresis loop, which is typical of mesoporous materials containing slit-shaped pores formed between plate-like reaction products. The hydrothermal treatment substantially increased the specific surface area from 10 755 to 170 260 cm^2^ g^−1^ and pore volume from 0.0397 to 0.0654 cm^3^ g^−1^, while dramatically reducing the average pore diameter from 95.5 to 12.7 nm. The shift toward smaller and more uniformly distributed mesopores indicates extensive microstructural refinement and accelerated formation of highly crystalline hydrated phases in addition to zeolitic networks.^45^ Hydrothermal curing at 170 °C for 5 h enabled the AAS paste to achieve a compressive strength of 63 MPa, corresponding to approximately 86% of the 28-day ambient-cured strength (73.1 MPa). Considering the substantial reduction in curing time from 28 days to only 5 h, this result demonstrates the exceptional effectiveness of hydrothermal treatment in accelerating slag activation and reaction-product formation. The pore structure analysis supports this observation, as hydrothermal curing transformed the adsorption behavior from Type III to Type IV/H3, while reducing the dominant pore diameter from 95.5 to 12.7 nm and increasing the specific surface area from 10 755 to 170 260 cm^2^ g^−1^. These changes indicate significant pore refinement and the formation of a highly developed mesoporous network, which contributed to the rapid development of mechanical strength.^34,56^

The microwave-cured AAS paste (450 W/10 min) again exhibited a Type III isotherm, suggesting that the pore system remained dominated by relatively large mesopores. The pore diameter increased markedly to 162.7 nm, while the pore volume decreased to 0.0186 cm^3^ g^−1^ and the surface area increased moderately to 28 432 cm^2^ g^−1^. The rapid evolution of water vapor during microwave irradiation likely generated larger capillary voids and coarser pores within the matrix. Consequently, the compressive strength reached only 39.2 MPa, substantially lower than those obtained under normal and hydrothermal curing. The coexistence of large pore diameters and lower mechanical strength confirms that rapid microwave heating, although highly effective for early strength development, may induce localized pore coarsening and partial disruption of the developing gel network.^55^ The influence of different curing regimes on the pore structure evolution of AAS is schematically illustrated in Fig. 11.

**Fig. 11:**
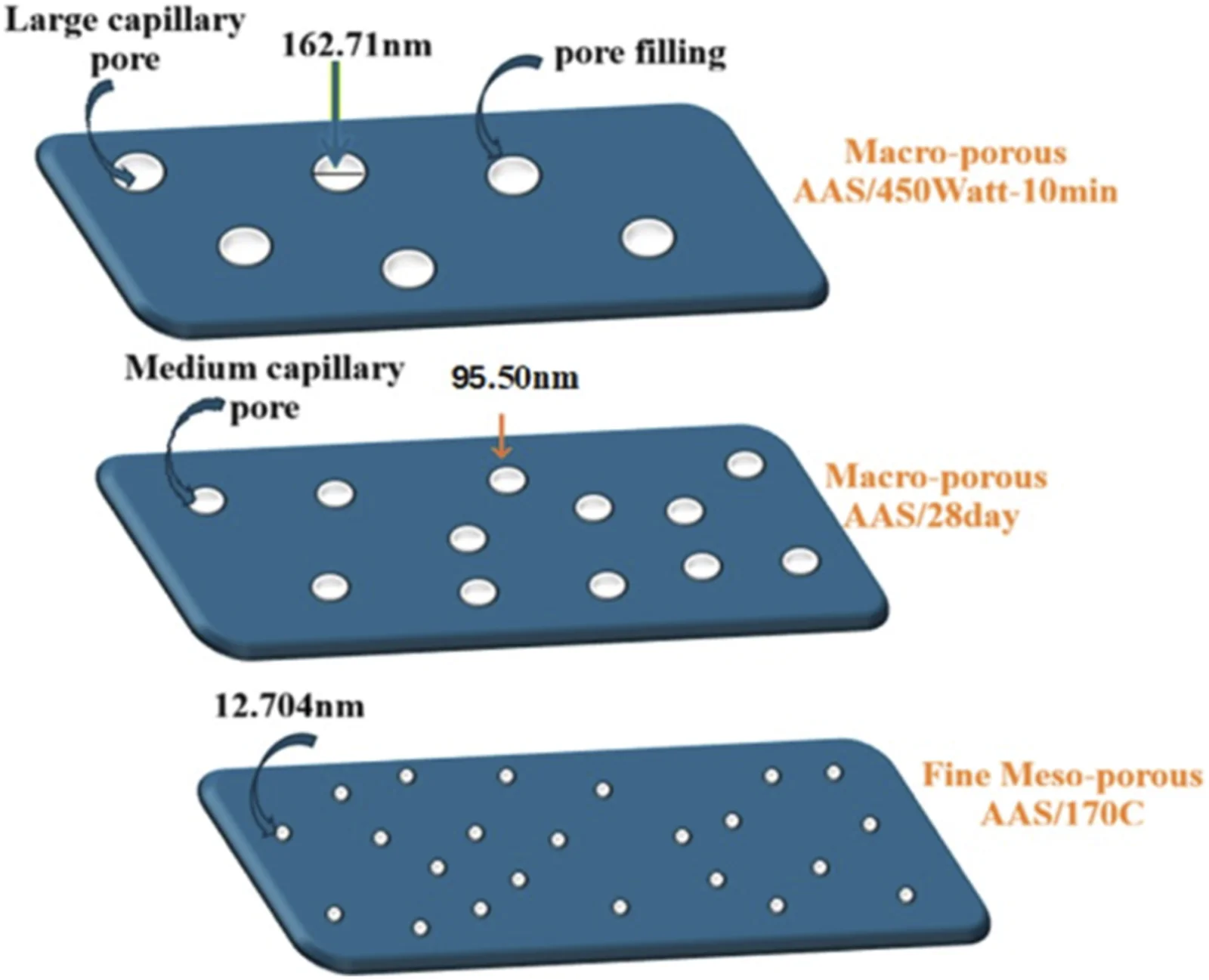
Schematic representation illustrating pore refinement of fabricated AAS under different curing regime.

A similar trend was observed for AAS-20BT. Under normal curing, the material displayed a Type III isotherm with a pore diameter of 35.6 nm, a pore volume of 0.0241 cm^3^ g^−1^, and a surface area of 29 767 cm^2^ g^−1^. Compared with the reference AAS paste, basalt incorporation reduced the average pore diameter and increased the specific surface area, indicating modifications in the packing characteristics and reaction kinetics of the binder system. Nevertheless, the corresponding compressive strength after 28 days was only 49.4 MPa, reflecting the lower reactivity of basalt relative to GGBFS and the consequent reduction in the amount of strength-contributing reaction products. Under hydrothermal curing (170 °C/5 h), AAS-20BT exhibited the most significant refinement of its pore structure, as evidenced by the transformation of the adsorption isotherm from Type III to Type IV with an H3 hysteresis loop, characteristic of well-developed mesoporous materials. This microstructural evolution was accompanied by a substantial increase in specific surface area to 397 290 cm^2^ g^−1^ and total pore volume to 0.1168 cm^3^ g^−1^, together with a marked reduction in the dominant pore diameter from 35.6 nm to 12.7 nm. Such pore refinement indicates accelerated dissolution of slag/basalt precursor and enhanced precipitation of reaction products under autoclaving conditions. The development of a highly connected mesoporous network is consistent with the XRD results, which confirmed the formation of abundant crystalline hydrates and zeolite-like phases, including phillipsite and stellerite-Na. These microstructural changes contributed directly to the improved mechanical performance, with the compressive strength increasing from 49.4 MPa under normal curing to 56.0 MPa after only 5 h of hydrothermal treatment, corresponding to an enhancement of 13.4%. Considering the substantial reduction in curing duration, this result highlights the remarkable efficiency of hydrothermal curing in promoting reaction kinetics, pore structure refinement, and matrix densification, ultimately leading to enhanced strength development.^37^ For microwave-cured AAS-20BT, the Type III isotherm reappeared, with a pore diameter of 95.5 nm, a pore volume of 0.0136 cm^3^ g^−1^, and a surface area of 19 984 cm^2^ g^−1^. Although microwave curing enabled rapid hardening and produced a compressive strength of 37 MPa after only 10 min, the relatively large pore size indicates that the pore structure remained coarser than that obtained under hydrothermal or prolonged ambient curing. The lower strength is therefore consistent with the presence of larger mesopores and reduced structural refinement.^57,58^

### 3.4. Phase compositions analysis

#### 3.4.1. X-ray diffraction analysis

Powder XRD was employed to ascertain both the phase composition of the solid powders and the successful synthesis of the assembled components.^59^Fig. 12 illustrates the XRD patterns of AAS and AAS-20BT binders cured under normal conditions (28 days), hydrothermal treatment (170 °C/5 h), and microwave irradiation (450 W/10 min). Generally, the XRD patterns of all composites exhibit a dominant broad halo within the 2*θ* range of 25–35°, which is attributed to the presence of poorly crystalline/amorphous reaction products, including sodium aluminosilicate hydrates (NASH) and calcium aluminosilicate hydrate gels that contribute to strength development. This broad hump is superimposed with distinguishable crystalline peaks corresponding to phases such as calcium silicate hydrates (C-S-H), hydrotalcite, calcium aluminosilicate hydrates (C-A-S-H), calcium aluminate hydrates (C-A-H), calcite, akermanite, albite, phillipsite, and stellerite-Na.

**Fig. 12:**
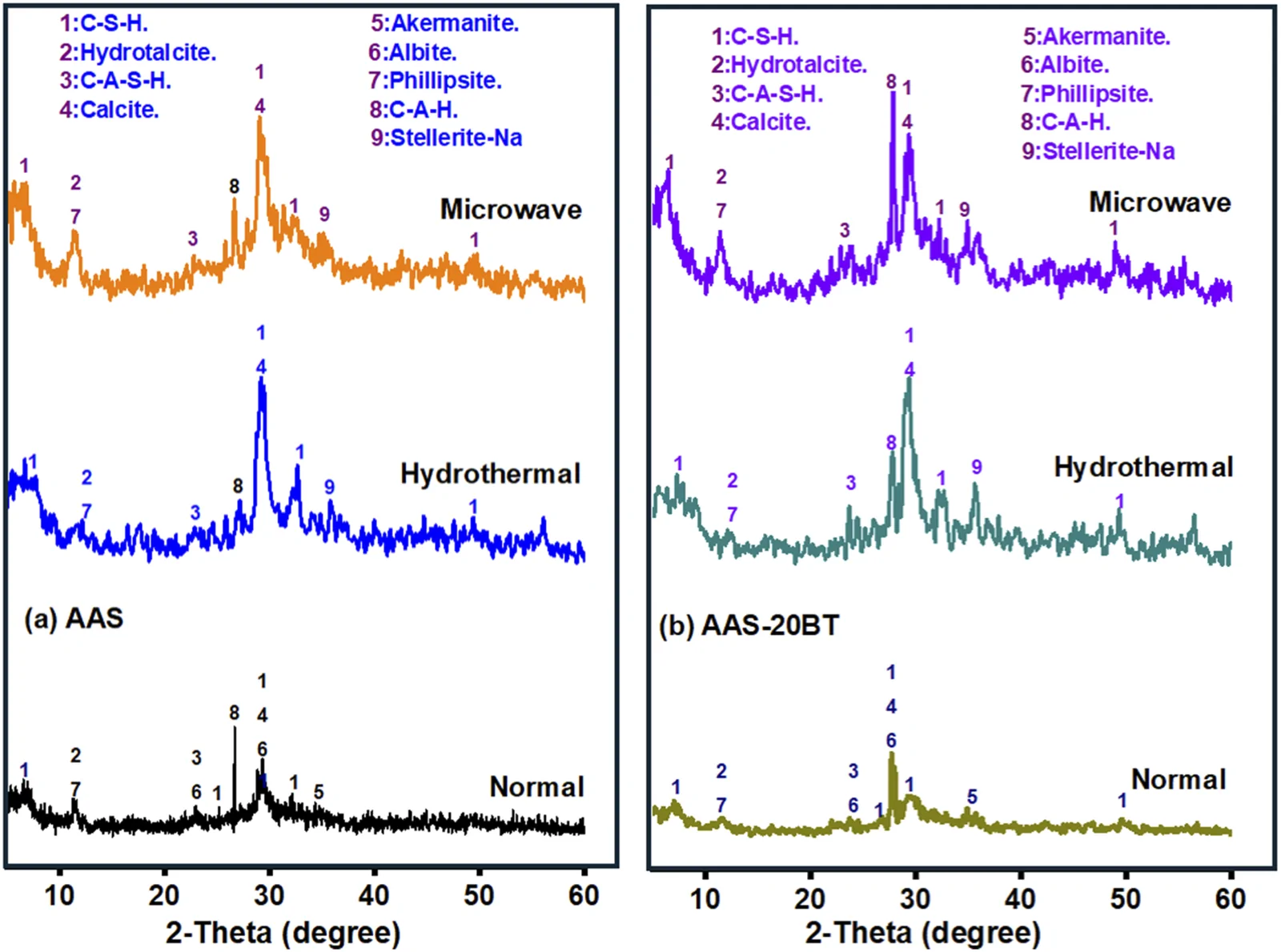
XRD-patterns of AAS and AAS-20BT geopolymers prepared under: (a1 and a2) ambient curing, (b1 and b2) autoclaving curing and (c1 and c2) microwave curing.

As clarified in Fig. 12, the XRD patterns of the ambient-cured specimens reveal the presence of predominantly amorphous reaction products, mainly calcium silicate hydrate (C-S-H; PDF# 00-003-0548, 2Ca_3_Si_2_O_7_·3H_2_O) and calcium aluminosilicate hydrate (C-A-S-H; PDF# 01-085-8414, Ca_2_Al(OH)_6_(AlSi(OH)_8_)(H_2_O)), which are responsible for strength development in the alkali-activated matrix.^55^ In addition, several crystalline phases were identified, including hydrotalcite (Mg_6_Al_2_CO_3_(OH)_16_·4H_2_O; PDF# 00-051-1525) and calcite (CaCO_3_; PDF# 00-001-0628).^60,61^ The patterns also exhibit residual crystalline phases originating from the unreacted precursor materials, primarily akermanite (Ca_2_MgSi_2_O_7_, PDF# 01-079-2424) and albite (NaAlSi_3_O_8_; PDF# 01-089-6425), indicating the incomplete dissolution of some mineral constituents during the alkali-activation process. The presence of hydrotalcite is consistent with the Mg- and Al-bearing chemistry of the system, while the calcite reflection is attributed to carbonation processes during curing.^62^ On the other hand, hydrothermal and microwave curing induce partial ordering of the binders and amplify the crystalline signature. Compared to ambient-cured specimens, these patterns demonstrate sharper and more intense reflections related to C-S-H and C-A-H.^63,64^ Moreover, new distinguishable peaks are observed, associated with zeolitic-like phases, specifically phillipsite, PDF#01-086-3924, K_1.8_Ca_1.2_(Al_5_Si_11_O_32_)(H_2_O)_14.36,_ and stellerite-Na, PDF#00-026-1382, Na_2_(Al_2_Si_7_)O_18_·7H_2_O. The intensification of these peaks and the appearance of zeolitic frameworks confirm the reorganization of the aluminosilicate gel network under accelerated curing conditions. This transformation indicates an enhanced dissolution of unreacted particles of aluminosilicate precursors and increased pozzolanic activity, which clarifies the role of these curing techniques in enhancing the compressive strength compared to ambient-cured ones.^65^ As clarified in Fig. 12, residual akermanite and albite phases were detected in the ambient-cured specimens, indicating incomplete dissolution of some precursor minerals during alkali activation. In contrast, these phases were not observed in the hydrothermal-cured specimens, suggesting enhanced dissolution of the basalt-derived minerals under elevated-temperature curing. The disappearance of these residual crystalline phases, together with the increased formation of C-S-H, C-A-S-H, and C-A-H reaction products, provides evidence of a higher reaction extent and improved microstructural development in the hydrothermally cured systems.

#### 3.4.2. FTIR analysis

As illustrated in Fig. 13, the FTIR spectra confirm the presence of a wide range of functional groups within both the AAS and AAS-20BT specimens subjected to ambient curing for 28 days, thermal curing at 170 °C/5 h, and microwave curing at 450 W/10 min. Regardless of the curing regime, all investigated specimens exhibit distinct transmittance bands distributed across specific vibrational regions. The bands located at 400–500, 600–800, and 900–1000 cm^−1^ are respectively attributed to Si–O–Si bending vibrations, symmetric stretching of Si–O–Al linkages, and asymmetric stretching of Si–O–Si(Al) frameworks. Furthermore, the bands identified at 1400–1600 cm^−1^ and 3500 cm^−1^ correspond to the carbonate-related bands and the symmetric stretching of hydroxyl groups, respectively.^66^ In comparison with the ambient-cured samples (AAS/28 days and AAS-20BT/28 days), the spectra for the thermally and microwave-cured specimens reveal a noticeable enhancement in band intensity accompanied by slight shifts toward higher wavenumbers within the Si–O–Al and Si–O–Si(Al) regions.^67^ These spectral modifications are indicative of a more advanced polymerization process of the silicate network and suggest the effective incorporation of reactive species into the aluminosilicate framework. Furthermore, the prominence of the O–H stretching bands reflects the generation of additional water-rich hydration products, which contribute to the evolution and stabilization of the geopolymeric matrix.^68^ The FTIR spectra provide clear evidence of the pronounced impact of accelerated curing on the structural maturation of the AAS pastes. Exposure to both hydrothermal conditions at 170 °C and microwave irradiation leads to sharper and more intense absorption bands, indicating an accelerated progression of geopolymerization and hydration reactions. The enhanced definition of the characteristic Si–O–T asymmetric stretching band signifies the development of a more ordered and crystalline aluminosilicate framework. The AAS-20BT samples exhibit markedly higher band intensities and sharper spectral features across all vibrational regions compared to the plain AAS samples, reflecting a more extensive structural reorganization. This advanced degree of network development contributes to a highly refined and interconnected microstructure enriched with active functional sites, which are directly linked to the enhanced performance of the material.^68–70^

**Fig. 13:**
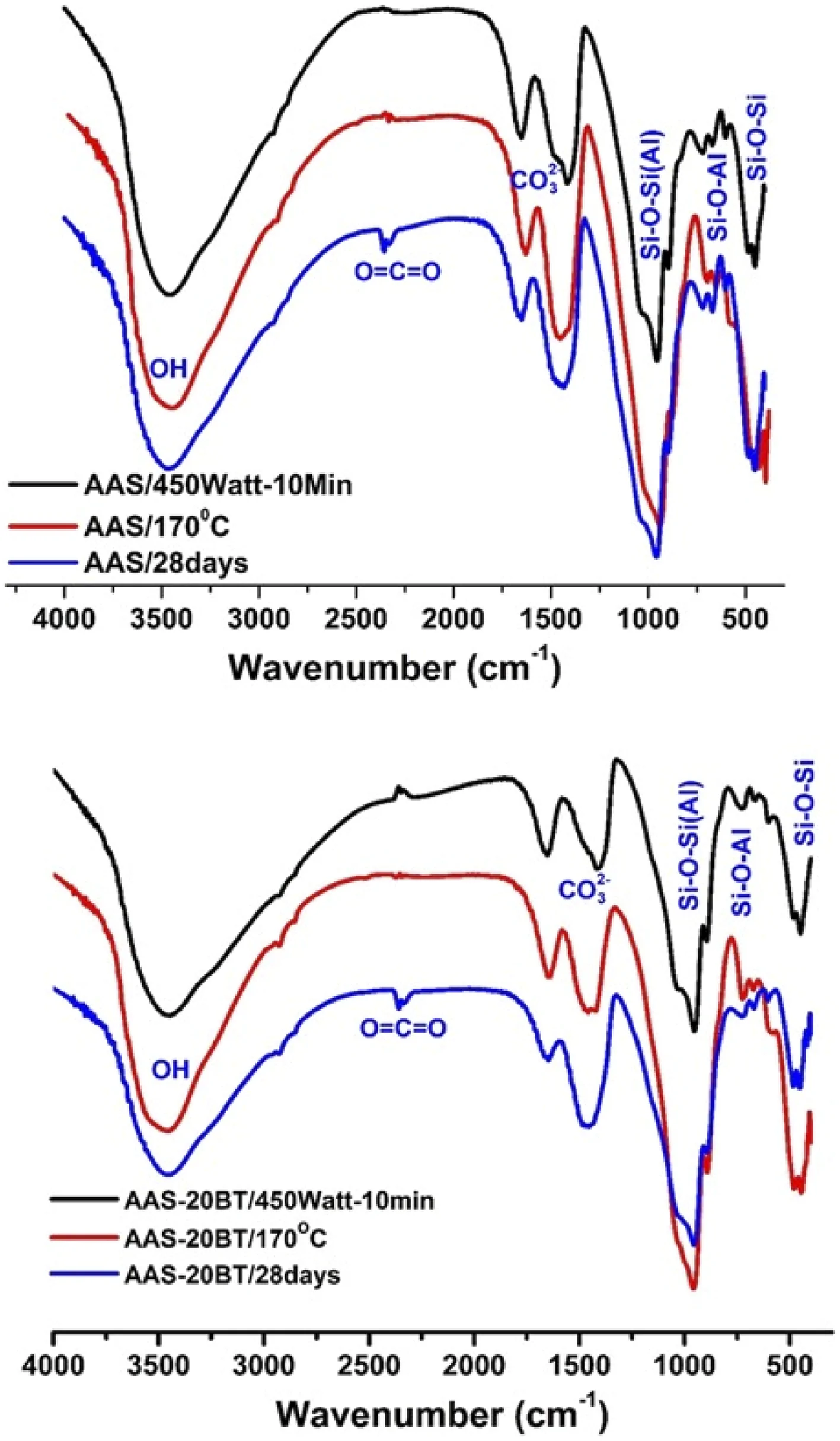
FTIR-spectra of AAS and AAS-20BT pastes prepared under different curing conditions.

### 3.5. Morphology and microstructure

The impact of the curing conditions on the developed composites AAS and AAS-20BT on the morphology of the strength-giving-phases and microstructure of the geopolymeric matrix was investigated using SEM/EDX. The SEM images show that the curing regime influences the transformation of GGBFS- and BT-containing precursors into a mechanically efficient gel network by affecting the reaction extent, pore refinement and microcrack formation. Under normal curing, Fig. 14a1 shows that AAS is composed of a compact structure of amorphous/semi-crystalline C-A-S-H, resulting from dissolution/condensation/polymerization of GGBFS.^71,72^ Such a phase fills the interparticle voids/pores, forming a continuous binding network, consistent with its relatively high compressive strength (59 MPa).^73^ In the AAS-20BT system, the SEM image in Fig. 14a2 reveals the formation of a less compact and heterogeneous microstructure composed of partially reacted BT particles engulfed in gel products, which is in line with its low compressive strength (49.5 MPa) relative to the AAS specimen. This observation can be attributed to the low pozzolanic activity of the BT under normal curing conditions,^37^ where BT acts as a filler and serves as nucleation seeds rather than a highly reactive aluminosilicate source.^74,75^ Also, the dilution of highly reactive GGBFS with BT leads to a reduction in the quantity of the formed binding gel.^74,76^ This interpretation is supported by EDX analysis, where normally cured AAS/28 days (Fig. 15a1 and Table 4) exhibited Ca/Si, Si/Al and Ca/Al ratios of 1.23, 1.69 and 2.09, respectively, indicating the formation of C-A-S-H binding phases.^77,78^ The EDX analyses were conducted as area measurements over the entire SEM image field rather than as localized spot analyses. Consequently, the reported elemental compositions represent average values across representative microstructural regions. It should be noted that EDX provides semi-quantitative compositional information and is therefore used only as supportive evidence alongside XRD, FTIR, BET/BJH, SEM, and compressive-strength results. The assessment of reaction development and matrix densification is based on the collective interpretation of these complementary characterization techniques rather than on EDX data alone. In the case of AAS-20BT/28 days (Fig. 15b1 and Table 4), these ratios increased to 1.40, 3.09 and 4.33, respectively. The high Ca/Si and Ca/Al ratios indicate a localized precipitation of calcium-rich strength-giving-phases, which are mainly derived from the dissolution of GGBFS. Simultaneously, the relatively low dissolution of BT under normal curing conditions may limit the release and incorporation of Al into the formed binding gel. This causes the analysed area to appear relatively enriched in Ca with respect to Al. On the other hand, the high Si/Al ratio indicates silica-rich aluminosilicate regions and partially reacted BT particles. This supports the discussion illustrated above; the BT acts as a filler and nucleation center, while its low pozzolanic activity, which results in incomplete activation, hinders the formation of continuous/homogenous load-bearing networks.

**Fig. 14:**
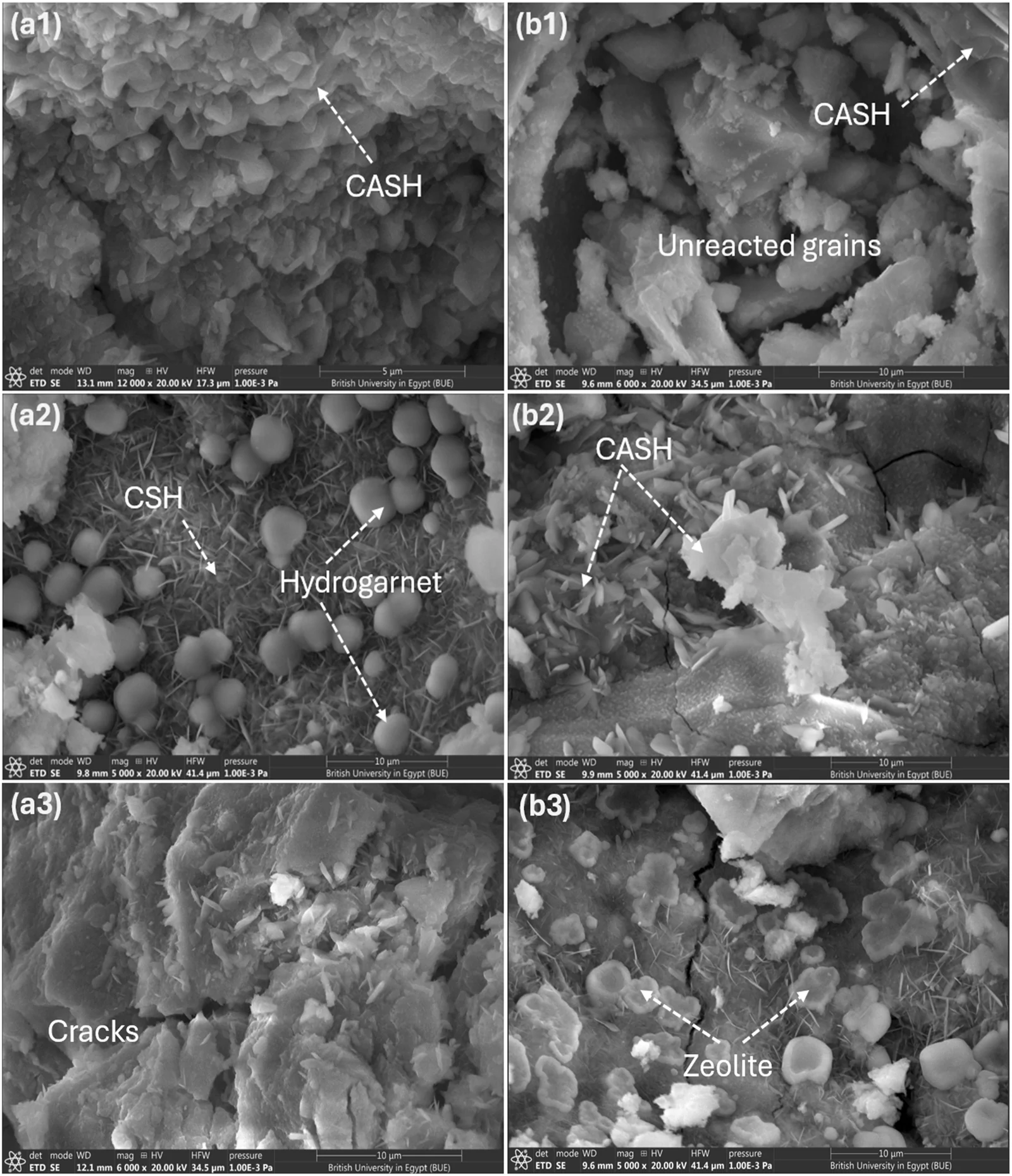
SEM images of (a1) AAS/28d, (a2) AAS/170 °C/5 h, (a3) AAS/450 W/10 min, (b1) AAS-20BT/28d, (b2) AAS-20BT/170 °C/5 h, and (b3) AAS-20BT/450 W/10 min.

**Fig. 15:**
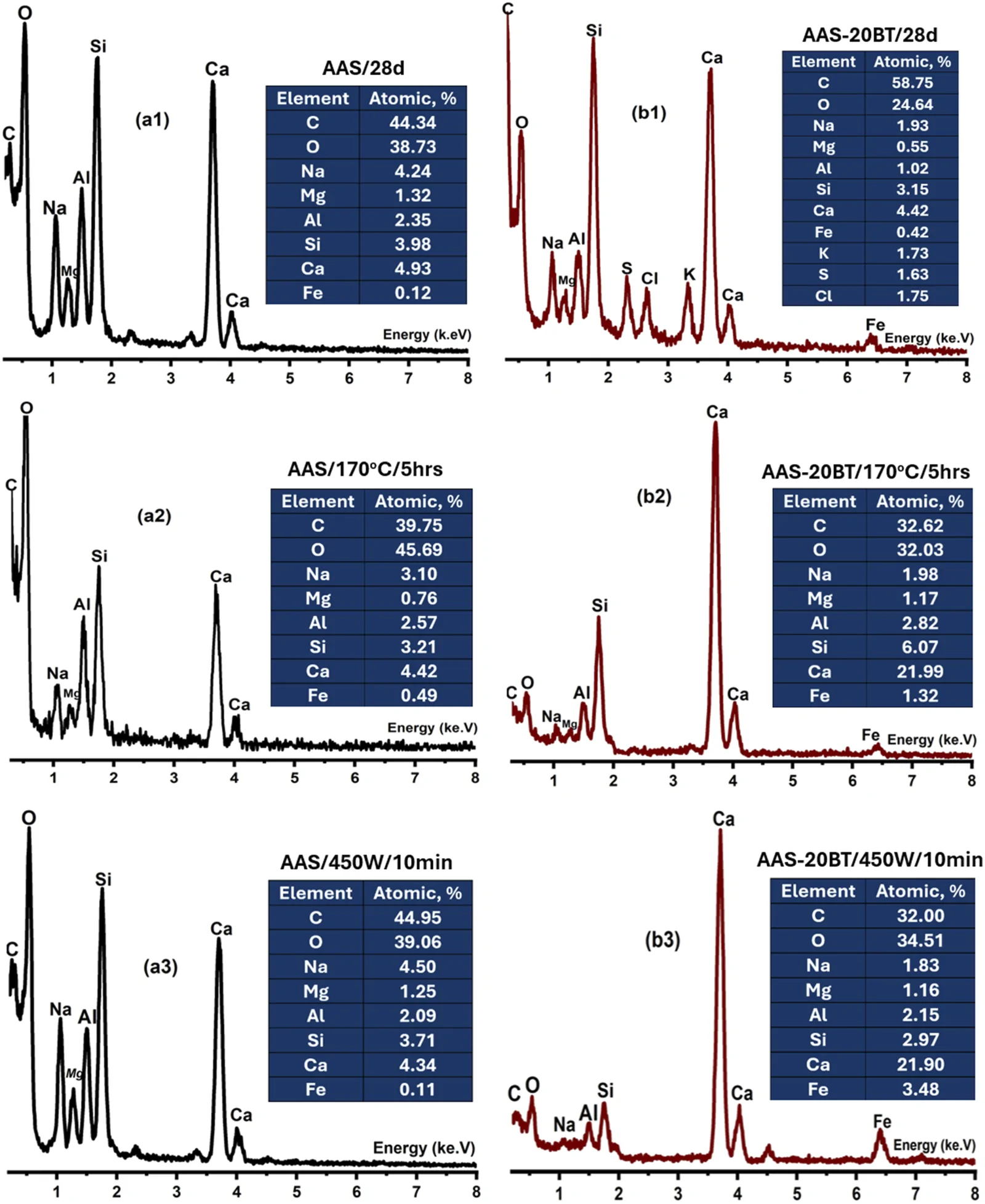
EDX-spectra of (a1) AAS/28 days, (a2) AAS/170 °C/5 h, (a3) AAS/450 W/10 min, (b1) AAS-20BT/28 days, (b2) AAS-20BT/170 °C/5 h, and (b3) AAS-20BT/450 W/10 min.

**Table 4:** EDS analysis of AAS and AAS-20BT pastes under different curing regimes (atomic, %)

Composite	Ca	Si	Al	Na	Ca/Si	Si/Al	Ca/Al	Na/Al
AAS/28 days	4.93	3.98	2.35	4.24	1.23	1.69	2.09	1.80
AAS/170 °C/5 h	4.42	3.21	2.57	3.10	1.38	1.25	1.72	1.21
AAS/450 W/10 min	4.34	3.71	2.09	4.50	1.17	1.78	2.08	2.15
AAS-20BT/28 days	4.42	3.15	1.02	1.93	1.40	3.09	4.33	1.89
AAS-20BT/170 °C/5 h	21.99	6.07	2.82	1.98	3.62	2.15	7.80	0.70
AAS-20BT/450 W/10 min	21.90	2.97	2.15	1.83	7.37	1.38	10.19	0.85

Under hydrothermal curing at 170 °C for 5 h, the SEM images of AAS (Fig. 14a2) and AAS-20BT (Fig. 14b2) show the formation of a dense/refined microstructure composed of interlocking needle-like, plate-like and foil-like C-S-H/C-A-S-H. Besides these phases, globular/sub-spherical crystalline particles associated with the hydrogarnet zeolitic phase appear. These observations can be attributed to the internal autoclaving process, driven by steam pressure and elevated temperature, which accelerates the dissolution of unreacted GGBFS and enhances the contribution of BT-derived minerals by increasing their pozzolanic activity.^37^ The compact morphology observed after hydrothermal curing is consistent with the BET/BJH results, which reveal substantial pore refinement. Specifically, the dominant pore diameter (dpmax) decreased from 95.5 to 12.7 nm for AAS and from 35.6 to 12.7 nm for AAS-20BT compared with the corresponding normally cured samples, confirming the effectiveness of hydrothermal treatment in densifying the pore structure. The EDX analysis of AAS/170 °C/5 h (Fig. 15a2 and Table 4) reveals that the Ca/Si, Si/Al and Ca/Al ratios are 1.38, 1.25 and 1.72, respectively. The obtained Ca/Al ratio is compatible with the formation of Ca-rich C-S-H and C-A-S-H. The reduction in the Si/Al ratio relative to AAS/28 days demonstrates the role of hydrothermal curing in enhancing the dissolution of unreacted particles in GGBFS, suggesting effective incorporation of Al into the hydrated framework. For EDX analysis of AAS-20BT (Fig. 15b2 and Table 4), the high Ca/Si, Si/Al and Ca/Al ratios of 3.62, 2.15, and 7.80 indicate precipitation of Ca-rich aluminosilicate hydrates. Interestingly noticing that the Na/Al ratio decreases from 1.89 in AAS-20BT/28 days to 0.70 in AAS-20BT/170 °C/5 h. This reduction reflects the effective consumption and distribution of Na species within strength-giving phases, instead of remaining as loosely bound or residual alkali-rich domains. In the geopolymeric system, lower residual Na usually indicates more advanced geopolymerization, as Na species act as charge-balancers within the hydration product structure rather than accumulating as unreacted alkaline species.^79–81^ Thus, the low Na/Al in the hydrothermal-cured AAS-20BT confirms the formation of a chemically stabilized matrix.

On the contrary, microwave curing at 450 W/10 min produces a less favorable microstructure, as shown in Fig. 14a3 and b3. Despite microwave irradiation being highly efficient at accelerating the dissolution of aluminosilicate precursors *via* internal heating,^18,19^ the SEM images show a discontinuous hydration gel, a non-uniform distribution of load-bearing-capacity phases, and microcracks. The evaporation of water during microwave heating can induce internal stress and cause shrinkage, leading to microcracks and open pores, thereby destroying the microstructure.^55^ This microstructural sequence matches the increase in dpmax observed in BET/BJH results for microwave-cured AAS and AAS-20BT specimens compared with hydrothermal-cured ones; the dpmax increased to 162.71 and 95.5 nm, respectively, explaining the reduction in strength. From Fig. 15a3 and Table 4, the Ca/Si ratio of 1.17 for AAS, which is still in the range associated with the formation of C-A-S-H. The high Na/Al ratio of 2.15 indicates a high abundance of loosely bound sodium species, reflecting insufficient incorporation into the aluminosilicate structure and then incomplete charge balancing in the gel structure. This clarifies that the rapid microwave-induced reaction was not accompanied by complete structural reorganization of the binding phases. For AAS-20BT (Fig. 15b3 and Table 4), the high Ca/Si and Ca/Al of 7.37 and 10.19, respectively, show a localized Ca-rich hydrate with low Si and Al. This also indicates that limited BT dissolution produces insufficient Si and Al under microwave curing.

### 3.6. Cost analysis and environmental impact

Recent studies increasingly emphasize that the sustainability of alternative cementitious materials should be evaluated not only in terms of mechanical performance, but also by considering economic and environmental indicators such as material cost, embodied energy, and CO_2_ emissions. This broader assessment is particularly important when alternative binders or waste-derived materials require additional processing or thermal curing, since the associated energy demand can influence both production cost and environmental impact. In line with this approach, the present study compares the cost and CO_2_ emissions of AAS-20BT under different curing regimes, while accounting for both the constituent materials and the electricity required during curing.^82,83^

The economic and environmental performance of the AAS-20BT geopolymer was evaluated under normal, hydrothermal and microwave curing conditions and compared with ordinary Portland cement (OPC), as summarized in Table 5. The comparison was performed based on producing 1 ton of binder. For AAS-20BT, the precursor consisted of 800 kg GGBFS and 200 kg basalt, activated with 60 kg NaOH. For the thermally assisted curing routes, the electricity required for curing was additionally included in both the cost and CO_2_-emission calculations. For the environmental assessment, the embodied CO_2_ emission factors adopted for OPC, GGBFS, basalt, and NaOH were 944, 26.5, 7.65, and 1232 kg CO_2_ per t, respectively.^38,84–87^ The CO_2_ intensity of electricity was 0.55 kg CO_2_ per kWh.^58,88^ For the economic evaluation, unit prices of 90, 46, 3.84 and 408 $ per t were considered for OPC, GGBFS, basalt and NaOH, respectively, while the electricity price was taken as 0.044 $ per kWh.^89^ These factors were applied to material quantities and curing-energy requirements to estimate total CO_2_ emissions and production cost per ton for each investigated system.

**Table 5:** CO_2_-emission and cost analysis for preparing 1 ton of OPC and AAS-20BT

Composite	Ingredient	CO_2_-emission from each item (kg)	Total CO_2_-emission (kg)	Cost for each item ($)	Total cost ($)
OPC	1000 kg OPC	944	944	90	90
AAS-20BT normal curing	800 kg GGBFS	21.2	96.65	36.80	62.05
	200 kg basalt	1.53		0.77	
	60 kg NaOH	73.92		24.48	
AAS-20BT hydrothermal curing	800 kg GGBFS	21.2		36.8	
	200 kg basalt	1.53	152.20	0.77	66.48
	60 kg NaOH	73.92		24.48	
	Electricity	55.55		4.44	
AAS-20BT microwave curing	800 kg GGBFS	21.2	137.9	36.8	65.35
	200 kg basalt	1.53		0.77	
	60 kg NaOH	73.92		24.48	
	Electricity	41.25		3.3	

The OPC reference exhibited the highest environmental burden, with total CO_2_-emission of 944 kg CO_2_ per t, whereas AAS-20BT under normal curing generated only 96.65 kg CO_2_ per t. This corresponds to an approximately 89.8% reduction in CO_2_-emissions compared with OPC. Among the AAS-20BT constituents, NaOH was the main contributor to the environmental impact, accounting for 73.92 kg CO_2_ per t, or about 76.5% of the total emissions under normal curing. GGBFS and basalt contributed only 21.2 and 1.53 kg CO_2_ per t, respectively. This indicates that, without additional thermal curing, the geopolymer's environmental impact is primarily controlled by the alkaline activator.

The electricity demand for curing was estimated from the rated power and operating time of the selected equipment. For hydrothermal curing, an industrial autoclave with a 90 kW heater and a 20 horsepower (hp) circulation fan (14.92 kW) was considered. Based on a chamber volume of 7.07 m^3^, assuming 50% loading and an AAS-20BT density of about 2200 kg m^−3^, the batch capacity was estimated at 7.78 tons. Heating from 25 to 175 °C at 1 °C min^−1^ followed by 5 h holding resulted in an estimated electricity consumption of approximately 101 kWh per t. For microwave curing, treatment of 1 kg AAS-20BT at 450 W for 10 min corresponded to 75 kWh per t. No external curing energy was assigned to normal curing. These values were then multiplied by the electricity emission factor of 0.55 kg CO_2_ per kWh and the electricity price of 0.044 $ per kWh to calculate the environmental and economic contributions of curing. Hydrothermal curing increased total CO_2_-emissions to 152.20 kg CO_2_ per t because of the electricity required for autoclave operation. Electricity contributed 55.55 kg CO_2_ per t, representing about 36.5% of the total emissions of the hydrothermally cured material. Nevertheless, the total emissions remained approximately 83.9% lower than OPC. Microwave curing resulted in a total emission of 137.9 kg CO_2_ per t, including 41.25 kg CO_2_ per t associated with electricity consumption. Thus, electricity accounted for about 29.9% of the total environmental impact of the microwave-cured system. Total CO_2_-emissions were about 85.4% lower than OPC and about 9.4% lower than hydrothermal curing. This lower energy-related impact reflects the considerably shorter microwave treatment duration compared with the 5 h hydrothermal curing cycle.

A similar trend was observed regarding production cost. OPC had a total material cost of 90 $ per t, while normally cured AAS-20BT had the lowest estimated cost of 62.05 $ per t, a reduction of about 31.1% compared with OPC. Including the curing energy increased the cost of hydrothermally cured AAS-20BT to 66.48 $ per t. Electricity cost was only 4.44 $ per t, about 6.7% of the total cost, although its contribution to the carbon footprint was considerably greater. Microwave curing resulted in a total cost of 65.35 $ per t, including an electricity cost of 3.30 $ per t. Consequently, microwave curing was approximately 1.7% less expensive than hydrothermal curing.

## 4. Conclusion

The main objective of this study was the sustainable utilization of basalt (BT) and ground granulated blast furnace slag (GGBFS) in the development of green building materials with satisfactory mechanical performance. GGBFS was partially replaced with 5, 10, and 20 wt% BT, and the effects of different curing regimes, namely normal curing, hydrothermal curing, and microwave curing, were investigated. Based on the experimental results, the following conclusions can be drawn:

• The incorporation of BT into the alkali-activated slag (AAS) matrix slightly reduced workability, with a decrease of 11.31% observed for the AAS-20BT mixture. However, the inclusion of BT had no significant effect on the setting time of the composites.

• BT negatively affected the early-age compressive strength (1 day) under ambient curing conditions. Nevertheless, at later curing ages (28 days), BT exhibited a beneficial effect on compressive strength when used in combination with GGBFS. The 28-day compressive strengths of AAS, AAS-5BT, AAS-10BT, and AAS-20BT were 59.0, 61.5, 55.0, and 49.5 MPa, respectively. The AAS-5BT mixture achieved the highest compressive strength among all tested compositions.

• Hydrothermal curing at 170 °C for 5 h and microwave curing at 450 W for 10 min proved highly effective in accelerating strength development, where only 5 h of autoclave curing and 10 min of microwave curing were sufficient for the AAS-20BT composite to achieve compressive strengths of 56 MPa and 37 MPa, respectively. These findings demonstrate the potential of both curing techniques for the rapid production of high-performance precast alkali-activated materials while significantly reducing curing time and energy consumption.

• BET/BJH, XRD, FTIR, and SEM/EDX analyses demonstrated that accelerated curing methods significantly enhanced porous system, the reaction extent, phase development, and microstructural refinement of the alkali-activated basalt/slag composites. These curing regimes promoted the formation of strength-giving C-S-H, C-A-S-H, and zeolitic phases, increased crystallinity, and produced a denser matrix, ultimately resulting in improved compressive strength compared with ambient curing.

• The combined use of GGBFS and basalt is recommended for sustainable alkali-activated materials. A 5 wt% basalt replacement yielded the highest strength, while hydrothermal (170 °C/5 h) and microwave (450 W/10 min) curing enabled rapid strength development, making them promising options for efficient precast production.

## Conflicts of interest

There are no conflicts to declare.

## Data Availability

The data supporting the findings of this study are available from the corresponding author upon reasonable request.

## References

[cit1] Davidovits J. , Geopolymer Chemistry and Applications, Geopolymer Institute, 2008

[cit2] Komnitsas K., Zaharaki D. (2007). Geopolymerisation: A review and prospects for the minerals industry. Miner. Eng..

[cit3] Yu Y. (2025). *et al.*, Compressive strength prediction of fly ash/slag-based geopolymer concrete using EBA-optimised chemistry-informed interpretable deep learning model. Dev. Built Environ..

[cit4] Ahmad J. (2022). *et al.*, A comprehensive review on the ground granulated blast furnace slag (GGBS) in concrete production. Sustainability.

[cit5] Nawaz M., Heitor A., Sivakumar M. (2022). Development and evaluation of a novel geopolymer based on basalt rock waste and ground granulated blast furnace slag. Aust. J. Civ. Eng..

[cit6] Mao Q. (2022). *et al.*, Mechanism, characterization and factors of reaction between basalt and alkali: Exploratory investigation for potential application in geopolymer concrete. Cem. Concr. Compos..

[cit7] Mohsen A. (2023). *et al.*, Bio-mechanical efficacy for slag/fly ash-based geopolymer mingled with mesoporous NiO. Case Stud. Constr. Mater..

[cit8] Akturk B. , *et al.*, Utilization of basalt powder as geopolymeric binder, in International Conference on “Cement—Based Materials Tailored for a Sustainable Future”, 2021

[cit9] Mashout H. A. (2023). *et al.*, Performance of nano titania-reinforced slag/basalt geopolymer composites. J. Eng. Appl. Sci..

[cit10] Lei S. , et al., Lightweight Geopolymers from Microwave Curing of Basalt and Waste Glass Powder Mixtures, 2023, available at SSRN 4542409

[cit11] Balapanov B. (2024). *et al.*, Accelerated microwave curing of hybrid geopolymers with nano-silica for enhanced physico-mechanical properties. J. Sustainable Constr. Mater. Technol..

[cit12] Shen Y.-N. (2026). *et al.*, Strain-hardening alkali-activated basalt/slag composites with high strength and high ductility: Unraveling the role of waste basalt powder. Cem. Concr. Compos..

[cit13] Liu J. (2023). *et al.*, Study the mechanical properties of geopolymer under different curing conditions. Minerals.

[cit14] Patil A. A., Chore H., Dode P. (2014). Effect of curing condition on strength of geopolymer concrete. Adv. Concr. Constr..

[cit15] Liu Q. (2022). *et al.*, Alkali-hydrothermal activation of mine tailings to prepare one-part geopolymer: Activation mechanism, workability, strength, and hydration reaction. Ceram. Int..

[cit16] Lee N., Khalid H. R., Lee H.-K. (2016). Synthesis of mesoporous geopolymers containing zeolite phases by a hydrothermal treatment. Microporous Mesoporous Mater..

[cit17] Balapanov B. (2024). *et al.*, Accelerated microwave curing of hybrid geopolymers with nano-silica for enhanced physico-mechanical properties. J. Sustainable Constr. Mater. Technol..

[cit18] Yang Y. (2023). *et al.*, Microwave-thermal-assisted curing method on geopolymer preparation from Panzhihua high-titanium slag by alkali activation. Constr. Build. Mater..

[cit19] Rashad A. M. (2025). *et al.*, Egyptian basalt powder as a fortifier for improved performance and sustainability of alkali-activated slag cement. Sci. Rep..

[cit20] Liu J. (2025). *et al.*, Investigation of Mechanical Properties of Recycled Aggregate Concrete Incorporating Basalt Fiber, Copper Slag, and Ground Granulated Blast Furnace Slag. Buildings.

[cit21] Sadrmomtazi A., Tahmouresi B., Saradar A. (2018). Effects of silica fume on mechanical strength and microstructure of basalt fiber reinforced cementitious composites (BFRCC). Constr. Build. Mater..

[cit22] Jing Z. (2008). *et al.*, Influence of tobermorite formation on mechanical properties of hydrothermally solidified blast furnace slag. J. Mater. Sci..

[cit23] Sünbül Ş., Tortum A. (2025). Strength and Microstructural Analysis of Nano Powder and Basalt Fibre Reinforced RCC Pavement Concrete. Gazi Univ. J. Sci. Part A:Eng. Innovation.

[cit24] Aschoff J. (2024). *et al.*, Effect of Microwaves on the Rapid Curing of Metakaolin-and Aluminum Orthophosphate-Based Geopolymers. Materials.

[cit25] Ramadan M. (2022). *et al.*, Valorization of hazardous glass wastes via geopolymer production utilized in gamma ray shielding applications: a comparative study with Portland cement. Radiat. Phys. Chem..

[cit26] Sayed D. (2022). *et al.*, Synergetic impacts of mesoporous α-Fe2O3 nanoparticles on the performance of alkali-activated slag against fire, gamma rays, and some microorganisms. J. Build. Eng..

[cit27] Mohsen A. (2022). *et al.*, Correlation between porous structure analysis, mechanical efficiency and gamma-ray attenuation power for hydrothermally treated slag-glass waste-based geopolymer. Case Stud. Constr. Mater..

[cit28] Ramadan M. (2023). *et al.*, Synergetic effects of hydrothermal treatment on the behavior of toxic sludge-modified geopolymer: Immobilization of cerium and lead, textural characteristics, and mechanical efficiency. Constr. Build. Mater..

[cit29] Li Z. (2017). *et al.*, Effect of nano-titanium dioxide on mechanical and electrical properties and microstructure of reactive powder concrete. Mater. Res. Express.

[cit30] Ramadan M., El-Gamal S., Selim F. (2020). Mechanical properties, radiation mitigation and fire resistance of OPC-recycled glass powder composites containing nanoparticles. Constr. Build. Mater..

[cit31] Wetwet M. M. (2023). *et al.*, Multifunctional cementitious blends containing zirconia nanoparticles: mechanical characteristics, gamma attenuation behavior, and self-cleaning performance. J. Build. Eng..

[cit32] Lu G.-M. (2019). *et al.*, Experimental investigation on the effects of microwave treatment on basalt heating, mechanical strength, and fragmentation. Rock Mech. Rock Eng..

[cit33] Lei S. (2024). *et al.*, Effect of calcination temperature on the activity of basalt tailings for the lightweight geopolymer from microwave curing. Mater. Chem. Phys..

[cit34] Abd El-Lateef H. M. (2026). *et al.*, Toward circular economy: In-situ zeolitic mineral wool waste/slag-based geopolymers for sustainable construction and wastewater remediation. Dev. Built Environ..

[cit35] Designation A. , C191: Standard test method for normal consistency and setting time of Hydraulic Cement, Annual Book of ASTM Standards, 2008, pp. 172–174

[cit36] ASTM , A Standard test method for compressive strength of hydraulic cement mortars (using 2-in. or [50-mm] cube specimens), Annual Book of ASTM Standards, 2013, vol. 4, iss. 1, pp. 1–9

[cit37] Al-Aziz A. A. (2025). *et al.*, Mechanical performance and gamma attenuation capability of sustainable pozzolanic cement incorporating basalt under different curing regimes. Sci. Rep..

[cit38] Mohamed O. A. (2023). *et al.*, Impact of microporous γ-Al2O3 on the thermal stability of pre-cast cementitious composite containing glass waste. Constr. Build. Mater..

[cit39] Ramadan M. (2023). *et al.*, Enhancement of fresh and hardened properties of geopolymeric composite containing toxic lead sludge: A comparative study between the effect of superplasticizer and thermal treatment of sludge. J. Build. Eng..

[cit40] Habib A. O. (2021). *et al.*, Studying the impact of admixtures chemical structure on the rheological properties of silica-fume blended cement pastes using various rheological models. Ain Shams Eng. J..

[cit41] Mohsen A. (2022). *et al.*, Rheological behaviour, mechanical performance, and anti-fungal activity of OPC-granite waste composite modified with zinc oxide dust. J. Cleaner Prod..

[cit42] Refaie M. (2023). *et al.*, The Effect of Structural Stability of Chemical Admixtures on the NaOH Alkali-Activated Slag Properties. J. Mater. Civ. Eng..

[cit43] Chen X., Sutrisno A., Struble L. J. (2018). Effects of calcium on setting mechanism of metakaolin-based geopolymer. J. Am. Ceram. Soc..

[cit44] De Silva P., Sagoe-Crenstil K., Sirivivatnanon V. (2007). Kinetics of geopolymerization: Role of Al2O3 and SiO2. Cem. Concr. Res..

[cit45] Ramadan M. (2024). *et al.*, Developing green slag/bentonite-based geopolymers modified with meso-porous tungsten oxide: Zeolitic phases, mechanical performance and gamma-radiation mitigation. Appl. Clay Sci..

[cit46] Nath S. (2016). *et al.*, Microstructural and morphological evolution of fly ash based geopolymers. Constr. Build. Mater..

[cit47] Saludung A. (2023). *et al.*, Mechanical and microstructural evolutions of fly ash/slag-based geopolymer at high temperatures: Effect of curing conditions. Ceram. Int..

[cit48] Schade T., Bellmann F., Middendorf B. (2022). Quantitative analysis of C-(K)-ASH-amount and hydrotalcite phase content in finely ground highly alkali-activated slag/silica fume blended cementitious material. Cem. Concr. Res..

[cit49] Aziz I. H. A. (2023). *et al.*, Mechanical performance, microstructure, and porosity evolution of fly ash geopolymer after ten years of curing age. Materials.

[cit50] Nodehi M. (2022). *et al.*, The effect of curing regimes on physico-mechanical, microstructural and durability properties of alkali-activated materials: A review. Constr. Build. Mater..

[cit51] Amin M. (2024). *et al.*, Synthesis of Multifunctional Mesoporous Geopolymer under Hydrothermal Curing: High Mechanical Resistance and Efficient Removal of Methylene Blue from Aqueous Medium. Dev. Built Environ..

[cit52] Abubakri S. , Microwave Curing of Concrete Repair, Sheffield Hallam University, United Kingdom, 2018

[cit53] Chen L. , Effect of Densification and Post-densification Regime on the Microstructure and Strength of Microwave Cured Concrete, UCL (University College London), 2022

[cit54] Xuequan W., Jianbgo D., Mingshu T. (1987). Microwave curing technique in concrete manufacture. Cem. Concr. Res..

[cit55] Mohsen A., Amin M., Ramadan M. (2025). Synthesis of highly efficient microwaved geopolymer based on alkaline activation of slag/bentonite precursors: The correlation among hydraulic phases, porosity profile, and compressive strength. Constr. Build. Mater..

[cit56] Ramadan M. (2023). *et al.*, Exploration of mechanical performance, porous structure, and self-cleaning behavior for hydrothermally cured sustainable cementitious composites containing de-aluminated metakaolin waste and TiO2 nanoparticles. J. Mater. Res. Technol..

[cit57] Sayed D., Ramadan M., Mohsen A. (2025). Valorizing calcinated-bentonite in the production of hydrothermally cured geopolymers with high mechanical-efficacy. Process Saf. Environ. Prot..

[cit58] Refaat M. (2023). *et al.*, Utilization of optimized microwave sintering to produce safe and sustainable one-part alkali-activated materials. Sci. Rep..

[cit59] Sayed M. A. (2023). *et al.*, Low-cost fabrication and physicochemical characterization of ZnFe2O4 nanoparticles as an efficient multifunctional inorganic pigment. J. Coat. Technol. Res..

[cit60] Sayed D. G., Ramadan M., Mohsen A. (2025). Valorizing calcinated-bentonite in the production of hydrothermally cured geopolymers with high mechanical-efficacy. Process Saf. Environ. Prot..

[cit61] Hassan H. (2023). *et al.*, Development of green ternary-blended-geopolymers for multifunctional engineering applications. Constr. Build. Mater..

[cit62] Yang J., Li D., Fang Y. (2017). Synthesis of nanoscale CaO-Al2O3-SiO2-H2O and Na2O-Al2O3-SiO2-H2O using the hydrothermal method and their characterization. Materials.

[cit63] Tian H. (2021). *et al.*, Influence of foreign ions on calcium silicate hydrate under hydrothermal conditions: A review. Constr. Build. Mater..

[cit64] Mesecke K. , Understanding Calcium Silicate Hydrate Formation during the Hydrothermal Curing of Autoclaved Aerated Concrete. 2023

[cit65] Puertas F. (2011). *et al.*, A model for the CASH gel formed in alkali-activated slag cements. J. Eur. Ceram. Soc..

[cit66] Nguyen T. N. (2022). *et al.*, Alteration in molecular structure of alkali activated slag with various water to binder ratios under accelerated carbonation. Sci. Rep..

[cit67] Thang N. H., Thach B. K., Minh D. Q. (2021). Influence of Curing Regimes on Engineering and Microstructural Properties of Geopolymer-Based Materials from Water Treatment Residue and Fly Ash. Int. J. Technol..

[cit68] Dakhane A. (2017). *et al.*, Time, temperature, and cationic dependence of alkali activation of slag: insights from Fourier transform infrared spectroscopy and spectral deconvolution. Appl. Spectrosc..

[cit69] Li Z. (2020). *et al.*, Internal curing of alkali-activated slag-fly ash paste with superabsorbent polymers. Constr. Build. Mater..

[cit70] Zhang G.-Y. (2021). *et al.*, Effect of waste ceramic powder on properties of alkali-activated blast furnace slag paste and mortar. Polymers.

[cit71] Ramadan M. (2024). *et al.*, Upgrading physico-mechanical characteristics and bacterial resistivity for cementless pastes modified with ZnAl2O4 nano-spinel: A comparative study with ZnO. J. Build. Eng..

[cit72] Mohsen A. (2024). *et al.*, The impact of wurtzite and mesoporous Zn-Al-CO3 LDH on the performance of alkali-activated-slag: Setting times, compressive strength, and radiation attenuation. Constr. Build. Mater..

[cit73] Amin M. S. (2024). *et al.*, Evaluation of mechanical performance, corrosion behavior, texture characterization and aggressive attack of OPC-FMK blended cement pastes modified with micro Titania. Constr. Build. Mater..

[cit74] Dobiszewska M., Schindler A. K., Pichór W. (2018). Mechanical properties and interfacial transition zone microstructure of concrete with waste basalt powder addition. Constr. Build. Mater..

[cit75] Alzeer M. I. M., Cheeseman C., Kinnunen P. (2022). New synthetic glass-based supplementary cementitious materials derived from basalt composition. J. Build. Eng..

[cit76] Çelikten S., Baran B. (2024). A comprehensive assessment of mechanical and environmental properties of waste basalt powder-modified high strength mortars exposed to high temperature. Next Mater..

[cit77] Yang S.-Y. (2021). *et al.*, Incorporation of sodium and aluminum in cementitious calcium-alumino-silicate-hydrate C-(A)-SH phases studied by 23Na, 27Al, and 29Si MAS NMR spectroscopy. J. Phys. Chem. C.

[cit78] Li J. (2019). *et al.*, The chemistry and structure of calcium (alumino) silicate hydrate: a study by XANES, ptychographic imaging, and wide-and small-angle scattering. Cem. Concr. Res..

[cit79] Valizadeh Kiamahalleh M. (2025). *et al.*, Advanced characterization of ambient-cured geopolymer paste with delithiated β-Spodumene: effect of Na2SiO3–to–NaOH ratio on performance and microstructure. Mater. Struct..

[cit80] Mokhtari P. (2024). *et al.*, Investigation of geopolymer efflorescence durability problems: causes and possible solutions. J. Am. Ceram. Soc..

[cit81] Rowles M., O'connor B. (2003). Chemical optimisation of the compressive strength of aluminosilicate geopolymers synthesised by sodium silicate activation of metakaolinite. J. Mater. Chem..

[cit82] Shen Y.-N. (2026). *et al.*, Limestone Calcined Clay Cement-based High-Strength Engineered Cementitious Composites (LC3-HS-ECC): Material design and bond performance. J. Build. Eng..

[cit83] Zhang Z.-L. (2026). *et al.*, Beyond performance-cost trade-off: Engineered Geopolymer Composites (EGC) incorporating ultra-high-volume coal gangue wastes. J. Cleaner Prod..

[cit84] Ramadan M. (2023). *et al.*, Exploration of mechanical performance, porous structure, and self-cleaning behavior for hydrothermally cured sustainable cementitious composites containing de-aluminated metakaolin waste and TiO2 nanoparticles. J. Mater. Res. Technol..

[cit85] Abd El-Lateef H. M. (2026). *et al.*, Role of meso-porous MgAl-LDH nanorods in the hydrothermal alkali activation of slag binders: Mechanical integrity and thermal stability. J. Mater. Res. Technol..

[cit86] Peng Z. (2026). *et al.*, Carbon emission assessment of basalt fiber and basalt fiber-reinforced polymer (BFRP) reinforced/strengthened concrete structures. Structures.

[cit87] Oppon E. (2024). *et al.*, Towards sustainable food production and climate change mitigation: an attributional life cycle assessment comparing industrial and basalt rock dust fertilisers. Int. J. Life Cycle Assess..

[cit88] Refaat M. (2021). *et al.*, Minimizing energy consumption to produce safe one-part alkali-activated materials. J. Cleaner Prod..

[cit89] Abd El-Lateef H. M. (2026). *et al.*, Role of meso-porous MgAl-LDH nanorods in the hydrothermal alkali activation of slag binders: Mechanical integrity and thermal stability. J. Mater. Res. Technol..

